# Outputs of generative diffusion models are often unattributable

**DOI:** 10.1038/s41467-026-75667-5

**Published:** 2026-08-18

**Authors:** Zheng Dai, David K. Gifford

**Affiliations:** Computer Science and Artificial Intelligence Laboratory, Massachusetts Institute of Technology, Cambridge, MA USA

**Keywords:** Computer science, Information technology

## Abstract

Modern generative diffusion models work by replicating the statistical patterns of large training datasets. Developing a method to attribute generated outputs to influential training data would greatly advance our understanding of and ability to regulate these models, leading to much work towards this goal. But is this possible? Here, we show that models trained with enough data often generate samples that are unattributable. We establish this through a large-scale analysis of what-if scenarios, revealing that we can often omit any sample or creator from the training data without affecting a generated sample. Our study focuses on diffusion models, which has become the dominant model for generating audiovisual media, and is also prevalent in many scientific applications including protein structure modeling and therapeutic discovery. Central to our analysis is a model ablation methodology that allows efficient removal of training examples from a trained model without the need to retrain.

## Abstract (web summary)

The authors investigate the attribution of diffusion model outputs to training data and show that generated samples can become unattributable when models are trained on large datasets. They introduce a model ablation method that enables efficient removal of training examples without retraining.

## Introduction

Diffusion models^1,2^ have emerged as powerful tools for generating realistic synthetic samples from training data, and have achieved remarkable results in a wide array of applications including image generation^3,4^, video generation^5^, audio synthesis^6^, protein folding^7^, and therapeutic design^8^. These generative models are created by optimizing their ability to reconstruct corrupted samples from training datasets. The resulting models therefore depend on their training datasets, and have been likened to compressed representations of them.

Is it possible to attribute samples generated by these models to the samples they ingest during training? This capability would be invaluable to our understanding of these models, not to mention a variety of applications including machine unlearning^9^, data poisoning^10^, model interpretability^11^, fairness^12^, and privacy^13^. Furthermore, given the contemporary adoption of these models for creative and commercial purposes, attributability also carries ethical, policy, financial, and legal implications.

Here we show that attribution, characterized as the task of locating a part of the training data that can be held responsible for a generated sample, can become impossible if a model is trained on a sufficiently large corpus of data. A large training set is all that is needed to induce this phenomenon of unattributability. Our analysis is based on observing changes in model behavior, or lack thereof, upon omitting a part of the training set. The effects of such omissions are evaluated with ablation, a technique we introduce which circumvents the prohibitive costs of retraining while eliminating any causal contamination that would be introduced through approximate methods^11,14–16^, problems which would have rendered our study intractable at meaningfully large scales.

Our results are organized as follows:

- We start by introducing a causal counterfactual framework for studying attributability, ablation as a technique for computing counterfactuals, and the diffusion ensemble as an architecture that implements ablation (under the subheading We quantify attributability using counterfactuals).
- We then present our result showing that attributability decays (under the subheading Attributability decays as models are trained on more data) and can vanish (under the subheading Generated samples can be completely unattributable) as models are trained on increasing amounts of data under a variety of conditions and metrics. We provide further support for this relation by showing that similarity based attribution results in false attributions in large training data regimes (under the subheading Attribution decay makes attribution by similarity unreliable).

## Results

### We quantify attributability using counterfactuals

**Fig. 1:**
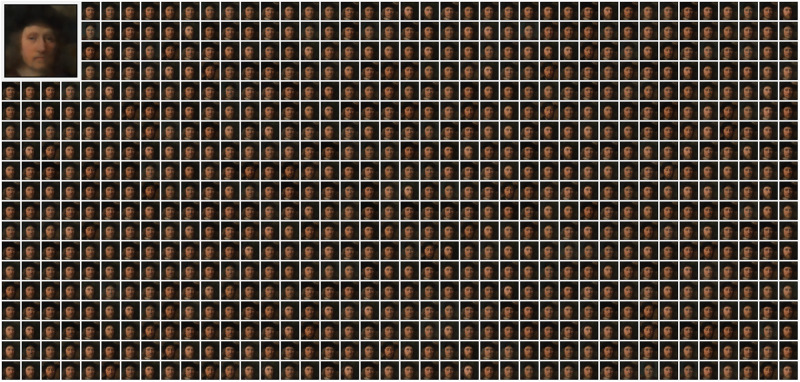
A generated image and its counterfactuals. On the top left we present an image generated by a model trained on public domain artwork created by 744 artists. Alongside are shown all the images that would have been generated had any one of the 744 artists been omitted from the training set. This set of images is created through an ablation based methodology, which allows us to circumvent the need to train 744 new models to produce these 744 counterfactual images.

**Fig. 2:**
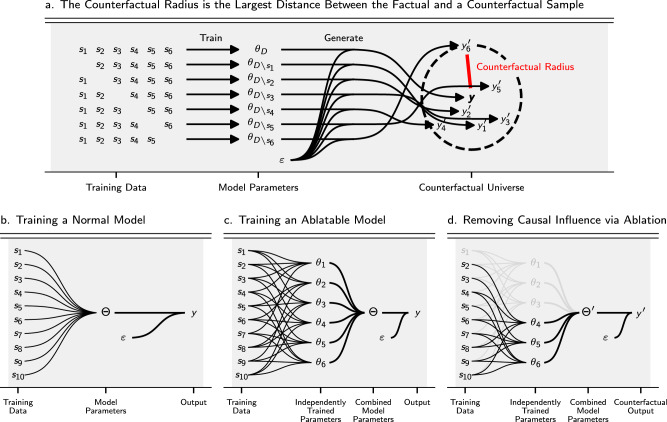
Counterfactual Radiuses quantify attributability and can be computed efficiently using ablatable models. **a** We use counterfactuals to quantify attributability. Conceptually, we consider each possible training set that can be obtained by omitting one unit of training data *s*_*i*_ from the full training set *D*. Each training set *D\s*_*i*_ can be used to train a model *θ*_*D\si*_, which can then be combined with exogenous factors *ε* to generate a set of counterfactual samples *y’*_*i*_, which make up the counterfactual universe. The Counterfactual Radius (CR) is the largest distance between the factual sample *y* and an element of this universe. Calculating the CR this way by training all required models is very expensive. An ablatable model will allow us to disentangle the causal influence of training data without this expensive training step. **b** We illustrate how normally, an entire model *Θ* is simultaneously trained on all units of training data *s*_*i*_. Exogenous factors *ε* are then added to the model to produce an output *y*. In this case it is impossible to remove the causal influence of a given *s*_*i*_ without fully retraining *Θ* from scratch. **c** We illustrate our proposed ablatable model structure, where individual model components *θ*_*i*_ are trained independently on different subsets of the training data before being combined into an overall model *Θ*. **d** We illustrate how this structure allows us to remove all causal influence from *s*_*1*_ by ablating away the parameters belonging to *θ*_*1*_, *θ*_*2*_, and *θ*_*3*_. The resulting model *Θ*’ is not causally influenced by *s*_*1*_, so the sample *y’* created by it also carries no causal influence from *s*_*1*_ but retains influence from all other *s*_*i*_.

Our study focuses on the attributability of images generated by diffusion models. We quantify the attributability of a generated image by measuring the largest change we can induce in the image by omitting a unit of training data. We use multiple methods to measure change, and we use the term *unit* to generically denote a part of the training data, generalizing the notion of a single image to a collection of one or more images, for example the collection of all images made by a single creator. The precise definition will be made explicit when relevant. We refer to the original and the changed image as the factual and counterfactual samples respectively. We call the collection of all counterfactual samples obtainable from a single factual sample as its *counterfactual universe*. An example of a counterfactual universe is shown in Fig. 1, and the idea is diagrammatically illustrated in Fig. 2a. We define the *Counterfactual Radius* (CR) of a factual sample as the largest distance between a member of its counterfactual universe and that factual sample. The CR of a factual sample quantifies its attributability. If the radius is small, then the factual sample cannot be significantly altered by the omission of a unit of training data, indicating the factual sample’s causal independence from and its unattributability to any single unit of training data^17^.

Training data is omitted by systematically removing the parts of a model that have been exposed to the data we wish to omit, a process we call *ablation* and illustrate in Fig. 2b–d. The ablated model can then be used to generate counterfactual samples by reusing the same exogenous factors used in the generation of the factual sample, such as the prompt and the random Gaussian noise injected into the reverse diffusion sampling process. Ablation requires bespoke architecture, for which we developed the *diffusion ensemble*, a model architecture that can withstand ablation and operates similarly to standard diffusion models with similar performance. The effectiveness of the diffusion ensemble architecture also makes diffusion models an especially suitable class of models for this study over other classes of generative models. Details on ablation and diffusion ensembles, including comparisons to standard diffusion models, are provided in the Methods section.

### Attributability decays as models are trained on more data

We find that the more data a model is trained on, the less attributable its generated samples become, a phenomenon we henceforth refer to as *attribution decay*. Specifically, we discover an inverse power law between the training set size of a model, quantified as the number of images the model is trained on, and the mean CR of the samples it generates.

**Fig. 3:**
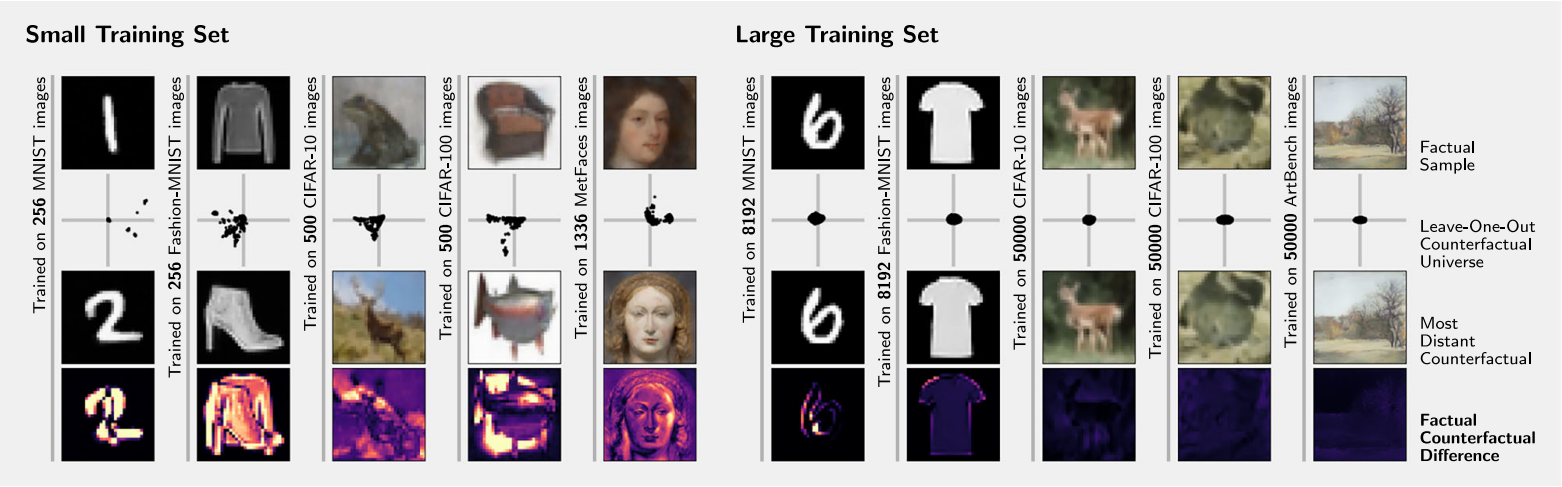
Samples generated by models trained on more data are less affected by training data omission. We illustrate the qualitative differences between counterfactual universes generated by ensembles trained on small and large datasets. The first row shows factually generated images. The second row shows pixel space principal component analysis (PCA) projections of the counterfactual universes of those images, revealing the geometric variance present within the counterfactual universe. Images are scaled to 256 × 256 × 3 before projection. Projections are all at the same scale and centered on the factual sample. The third row shows the furthest members in those counterfactual universes, measured geometrically. The final row shows heatmaps of the differences between the factual images and the furthest members of their counterfactual universes (first and third rows). Counterfactual universes cluster near factual images when training sets are large (right), but not when they are small (left).

**Fig. 4:**
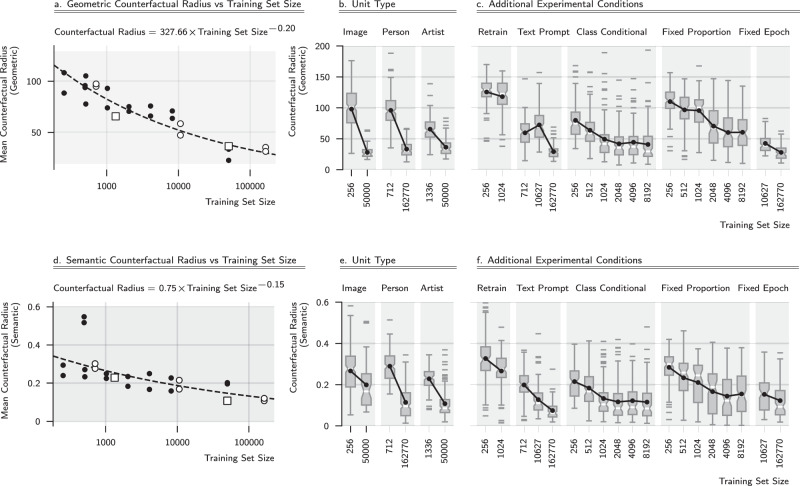
Attributability diminishes quantifiably as models are trained on more data. **a** Geometric CR is negatively related to training set size. We plot an ensemble’s mean CR against the number of images it was trained on. Images are scaled to 256 × 256 × 3 before distance measurements to allow comparison across image distributions. A fitted power law relation is given above the panel and plotted as a dashed line. Black circles denote ensembles where units consist of single images, white circles denote ensembles where units consist of all images of a single person, and white squares denote ensembles where units consist of all images created by a single artist. **b** We show how the distribution of geometric CRs shift as you move from the smallest training set size to the largest when restricted to a single definition for a unit of data (single image, single person, or single artist). Distributions are illustrated as box plots. The box itself spans the first to the third quartile, whiskers extend to either the furthest point or 1.5 times the interquartile range, whichever is shorter. Points beyond the whiskers are marked with horizontal lines. The median is marked with a white line and the notch indicates its 95% confidence interval (see Supplementary Information for details). The means of the distributions are traced out by solid black lines. **c** We show the distribution of CRs generated under additional experimental conditions. In order: when counterfactual samples are generated from retrained models rather than from ablated ensembles, when samples are generated from ensembles that condition on text prompts, when samples are generated from ensembles that condition on class labels, when samples are generated from ensembles where the number of units in the training sets are fixed to 96, and when samples are generated from ensembles trained for the same number of epochs. **d**–**f** Semantic CR is negatively related to training set size. The analysis is identical to what is presented in **a**–**c**, but CR is measured using a semantic distance instead of a geometric one. Attribution decay remains prominent.

Comprehensive experimental details are provided in the Supplementary Information. To summarize, we trained 24 diffusion ensembles on variably sized subsets (256 to 162,770 images) of 7 publicly accessible image datasets (MNIST^18^, Fashion-MNIST^19^, CIFAR-10, CIFAR-100^20^, CelebA^21^, MetFaces^22^, and ArtBench)^23^. To avoid confusion with the training datasets we derive from them, we will henceforth refer to these image datasets as image distributions. Mean CRs were calculated by generating 25–100 samples from each ensemble, calculating their CRs, and taking their arithmetic means. We present select counterfactual universes created in this process in Fig. 3 to provide visual intuition on how these counterfactual universes look across varying training set sizes and image distributions. To calculate CRs, image differences were quantified using both geometric distance as measured by the pixel level Euclidean distance, and semantic distance as measured by OpenCLIP^24^. Geometric distances on their own can be misleading when used to quantify image differences^25^, hence the need for additional metrics to support our results. We then fitted power laws between the mean CRs and training set sizes, which hold with *R*^2^ = 0.85 for geometric CRs and *R*^2^ = 0.59 for semantic CRs. The relations are provided in Fig. 4a, d respectively. The one-sided Wald test was used to establish that CRs and training set sizes are inversely related with significance (*p* < 10^−5^ for both relations).

### Attribution decays when attributing to people and artists

Attribution decay remains observable when we restrict the definition of a single unit of training data. For 16 of the 24 ensembles, each image is its own unit. For 6 of the 24 ensembles, which were trained on pictures of people, a unit consists of all images of a given person. For the remaining 2 ensembles, which were trained on artwork, a unit consists of all images created by a single artist. In all three cases the distribution of CRs conditioned on the largest training set size is significantly smaller than the distribution of CRs conditioned on the smallest training set size. The distributions are plotted in Fig. 4b, e. For both geometric and semantic CRs we observe drops with *p* < 10^−4^ for single image units, *p* < 10^−53^ for single person units, and *p* < 10^−22^ for single artist units. *P*-values are given by the one-sided Mann-Whitney U test.

This demonstrates that at large training set sizes it not only becomes infeasible to attribute generated images to training images, it also becomes infeasible to attribute generated people to the real people the model was trained on, and to attribute generated artwork to the artists the model was trained on.

### Attribution decay is not an artifact of ablation

To ensure that our findings are not an artifact of our ablation methodology, we reproduced our results at a small scale using more standard methodology, where counterfactuals are generated by models retrained from scratch without the omitted images rather than from ablated models. We took subsets of sizes 256 and 1024 of the MNIST image distribution and trained full leave-one-out fleets of diffusion models at each size, giving a total of 1282 diffusion models (1280 counterfactual and 2 factual). We sampled 100 counterfactual universes from each fleet and measured their geometric and semantic CRs. Significant drops in both geometric and semantic CRs were observed when the training set size was increased from 256 to 1024, consistent with attribution decay (*p* < 10^−4^ via the one-sided Mann-Whitney U test). The distributions are plotted in Fig. 4c for geometric CRs and in Fig. 4f for semantic CRs.

### Attribution decays in text and class conditioned models

To validate that attribution decay is also present in models that condition their sampling on text prompts, we trained a set of text prompt conditioned diffusion ensembles on the CelebA image distribution with training set sizes varying from 712 to 162,770. We sampled 100 counterfactual universes from each ensemble using a variety of prompts (details on prompt selection are provided in the Supplementary Information) and measured their geometric and semantic CRs. Significant drops in both geometric and semantic CRs were observed when the training set size was increased from 712 to 162,770, consistent with attribution decay (*p* < 10^−21^ via the one-sided Mann-Whitney U test). The distributions are plotted in Fig. 4c for geometric CRs and in Fig. 4f for semantic CRs.

Separately, we trained a set of class conditioned diffusion ensembles on the MNIST image distribution with training set sizes varying from 256 to 8192. These ensembles take one of 10 class labels as input and output images of that class. We generated 100 samples spread evenly across the different class labels for each ensemble and measured their geometric and semantic CRs. Significant drops in both geometric and semantic CRs were observed when the training set size was increased from 256 to 8192, consistent with attribution decay (*p* < 10^−13^ via the one-sided Mann-Whitney U test). The distributions are plotted in Fig. 4c for geometric CRs and in Fig. 4f for semantic CRs.

### Attribution decays when we fix proportion of omitted data

Increasing the training set size does not only affect the amount of data a model is trained on, it also shrinks the relative change in the training set caused by the omission of a single sample. This raises the possibility that attribution decay is driven by the shrinking difference between counterfactual and factual scenarios, rather than the increasing amount of data a model is trained on.

To rule out this possibility, we establish the presence of attribution decay in what we call a Fixed Proportion experimental condition where the proportion of data that gets removed in each counterfactual scenario was held constant while training set sizes were allowed to vary. Specifically, we randomly partitioned the MNIST image distribution into 96 units and trained diffusion ensembles on variably sized subsets of the partitioned dataset. We sampled 100 counterfactual universes from each ensemble and measured their geometric and semantic CRs. Significant drops in both geometric and semantic CRs were observed when the training set size was increased from 256 to 8192, consistent with attribution decay (*p* < 10^−16^ via the one-sided Mann-Whitney U test). The distributions are plotted in Fig. 4c for geometric CRs and in Fig. 4f for semantic CRs.

### Attribution decays when we fix training epochs

For most of our ensembles, we varied the number of epochs used for training so that ensembles trained on the same image distributions are trained for roughly the same number of iterations. The number of epochs each ensemble is trained for are reported in Supplementary Tables 2 and 3. This means that images in smaller training sets are seen more times than images in larger training sets during training.

To ensure that attribution decay persists even if each sample is processed the same number of times during training, we trained ensembles on subsets of size 10,627 and 162,770 from the CelebA image distribution for the same number of epochs. We sampled 100 counterfactual universes from each ensemble and measured their geometric and semantic CRs. Significant drops in both geometric and semantic CRs were observed when the training set size was increased from 10,627 to 162,770, consistent with attribution decay (*p* < 10^−12^ for geometric CRs and *p* < 0.002 for semantic CRs via the one-sided Mann-Whitney U test). The distributions are plotted in Fig. 4c for geometric CRs and in Fig. 4f for semantic CRs.

### Attribution decay is found with additional metrics

All the above analysis was repeated using two other image similarity metrics (LPIPS^26^ and DINOv2)^27^ to further ensure that our conclusions are robust to the choice of metric. Furthermore, we repeated our analysis of the Retrain, Class Conditional, and Fixed Proportion experimental conditions on a different image distribution (Fashion-MNIST). We found that attribution decay persists in this additional analysis with significance. The full analysis is presented in the Supplementary Information.

While the CR is the metric of primary interest, we also checked the relationship between training set size and alternative summaries of the counterfactual universe, such as the 25th, 50th, and 75th percentiles of distances between the counterfactual and factual samples. We find that a negative relation exists for these alternative summaries as well, although diminished for the lower quantiles. The full analysis is presented in Supplementary Fig. 6.

### Generated samples can be completely unattributable

Finding a sample with a CR of zero in a continuous setting is effectively a zero probability event due to uncontrollable exogenous factors that cause fluctuations in the counterfactual universe. However, zero CR samples can occur naturally if we move to a discrete setting. To verify the existence of such samples, we trained a diffusion ensemble on a set of binarized images taken from the MNIST image distribution where pixel values were rounded to either 0 or 1. We then generated and measured the CRs of 3731 samples from this ensemble, out of which we found 14 that had a CR of 0, confirming the existence of fully unattributable samples.

We note that a discretized setting is more realistic than a continuous one. Digital samples have finite bit representations, meaning that they exist in some finite and therefore discrete space. Images are usually encoded as grids of pixels, where pixel values are drawn from a finite pool of around 17 million RGB tuples.

### Attribution decay makes attribution by similarity unreliable

**Fig. 5:**
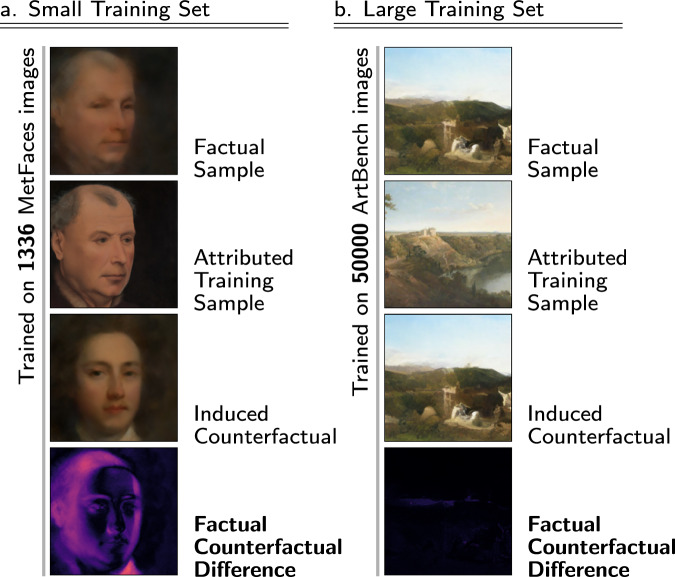
Omitting attributed data has a stronger effect when models are trained on less data. **a** The top panel shows an image generated by a model trained on 1336 artworks. We attribute it to the geometrically closest image in the training set, Man in Prayer, a 15th century painting by either Robert Campin or Rogier van der Weyden, shown in the second panel. Upon counterfactually omitting all artwork by Robert Campin from the training set, we find that the model generates a significantly different image, which is shown in the third panel, and whose difference from the original image is visualized in the fourth panel. **b** The top panel shows an image generated by a model trained on 50,000 artworks. We attribute it to the geometrically closest image in the training set, View of Castel Gandolfo, an 18th century painting by Thomas Jones, shown in the second panel. However, even after counterfactually omitting all artwork by Thomas Jones from the training set, we find that the image the model generates, which is shown in the third panel, is largely unchanged. The difference between the factual and counterfactual image is visualized in the fourth panel.

As further support for attribution decay, we find that the accuracy of attributions based on image similarity drops with increasing training set sizes. To determine the correctness of an attribution, we rely again on counterfactual analysis. The key idea is that a correct attribution must be contingent upon the generative model being trained on the attributed unit of training data. So, if we omit the attributed unit from the training set, the induced counterfactual sample cannot remain attributed to that same unit. If it does, then we can conclude that the attribution was incorrect. Therefore, by counting the number of times that an attribution remains unchanged upon omitting the attributed unit from the training data, and by adjusting for uncontrollable exogenous factors, we can obtain a *False Attribution Rate* (FAR). Details on this calculation are provided in the Methods section. An example of how a generated sample is affected by omitting the attributed sample is provided in Fig. 5.

**Fig. 6:**
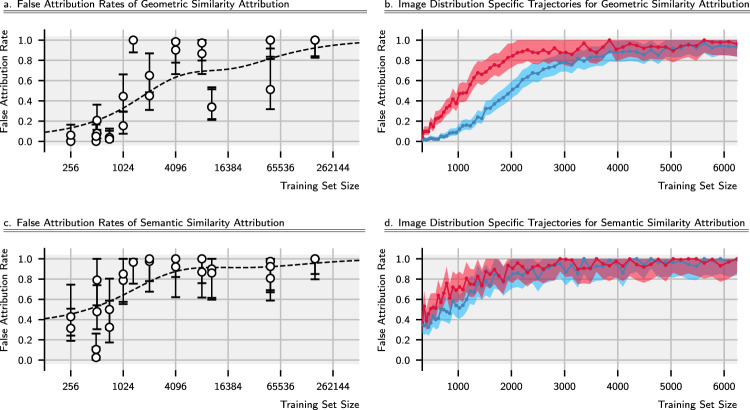
False attributions are prevalent in large training set regimes. **a** Evaluation of FARs for attribution based on geometric similarity. We show FARs evaluated on 24 ensembles. Clopper-Pearson 95% confidence intervals (*N* = 100) are indicated with error bars. Details regarding the calculation of the Clopper-Pearson confidence interval are given in the Methods section. A moving average of the FAR as a function of training set size is plotted as a dashed line. The moving average is computed via a Gaussian Kernel Regression, and details on the calculation are provided in the Supplementary Information. **b** We plot the FARs against training set size for attributions based on geometric similarity. Two curves are plotted, one based on ensembles trained on subsets of the MNIST image distribution (in blue), and one based on ensembles trained on subsets of the Fashion-MNIST image distribution (in red). Their Clopper-Pearson 95% confidence intervals (*N* = 1024) are also provided as bands around the plotted curves. False attributions increase near monotonically with training set sizes. **c**, **d** Evaluation of FARs for attribution based on semantic similarity. The analysis is identical to what is presented in **a**, **b**, but attribution is performed using a semantic distance as opposed to a geometric one. The effect of attribution decay remains prominent.

We estimated the FARs of attribution methods on 24 diffusion ensembles trained on variably sized subsets of the 7 image distributions discussed previously. We observed that ensembles trained on more data exhibit larger FARs. Here, attribution was performed using a straightforward nearest neighbor methodology where generated images are attributed to images in the training data that are most similar to them. As before, both geometric (Euclidean distance) and semantic (OpenCLIP) measures were used to quantify image similarity. Analyses with additional metrics yield similar results and are presented in the Supplementary Information. Each FAR was estimated using 100 samples, and are presented with confidence intervals in Fig. 6a, c.

Additionally, we evaluated the FARs of two sequences of 62 ensembles trained on subsets of the MNIST and Fashion-MNIST image distributions, which we plot in Fig. 6b, d These FARs were estimated from 1024 samples for each ensemble. We observe a near monotonic relation between the FARs and training set sizes in these curves once image distribution is removed as a confounder. While both curves are monotonic, they exhibit different rates of attribution decay, demonstrating that the exact decay characteristics can depend on image distribution.

## Discussion

Our findings provide a compelling case that attribution, the task of locating a unit of data within the training set that can be held responsible for a generated sample, is practically impossible on contemporary generative diffusion models. Attributability already decays significantly at scales of 10^4^ and 10^5^, and many commercially deployed models are trained on much larger datasets with up to ~10^9^ images^28^. Yet paradoxically, training data attribution is an active field of research where new and promising methods of attribution are continuously developed^29,30^. This paradox is resolved by noting that most contemporary methods of data attribution are not evaluated on a leave-one-out benchmark. Instead, they are typically evaluated by identifying and omitting large swathes of influential data.

We emphasize that our study focuses on leave-one-out style attribution, where the goal is to find a single unit of training data to which to attribute a generated sample. This differs from aggregate, subset-level, or approximate attribution methods, which are valuable for many applications. Our main findings do not preclude the ability to perform these other kinds of attributions, however they do show that further refinement along these lines will likely not yield methods that can perform attribution on the level of individual images, people, or artists.

Unattributability is an important consideration for the governance of generative models. A notable example is the commercial use of generative models trained on copyrighted material to produce market substitutes of said material. In this case, unattributability ostensibly provides a refutation of access^31^, a key element used in establishing infringement, thereby circumventing the intellectual property protections designed to limit such use as long as the harvest is conducted on a sufficient scale for attribution decay to manifest.

On the other hand, attribution decay means that models are naturally endowed with a privacy property that arises when they are trained on enough data. For example, our experiments show that generated images of people are often causally independent of the people who were used to train it, effectively preserving their privacy as long as sufficient data was used to train the generative model.

As a caveat, we note that since our analysis tracks aggregate model behavior, a model can potentially generate an attributable sample even if trained on vast amounts of data. It has previously been observed in the memorization literature that even productionized models trained on ~10^9^ samples can generate near identical copies of their training samples, albeit at a rate of around one in a million^32^. However, one could use the techniques introduced in this work to produce samples that are guaranteed to be unattributable: simply keep generating samples until a sample with a sufficiently low CR is generated. Our ablation based methods will allow one to compute CRs efficiently, and attribution decay will ensure that low CR samples are generated with reasonable frequency as long as the generating model was trained on enough data.

Our results are congruent with results in the memorization literature showing that the amount of copying from training data decreases when training set sizes are increased^33^. It is important to note that an absence of copying, which is detected using image similarities between generated samples and training samples and is used widely in the generative memorization literature as a measure of memorization, does not preclude the existence of other signals that may be used to attribute generated samples, which requires a counterfactual analysis like the one presented in this study. That said, given that we have established that large training set sizes, a factor that has been observed to reduce memorization, also reduces attributability, we might expect that other mitigating interventions in the memorization literature such as training data optimization and machine unlearning^34^ might also reduce a model’s propensity to generate attributable samples.

A possible exception is data deduplication, which is generally thought to reduce memorization^34^, but may improve attributability. We conjecture that attribution decay happens because features that are important to model behavior are distributively and redundantly encoded throughout its training set. This diffuse redundancy then permits us to omit parts of the training data without affecting the behavior of the model. It is only when training data is sparse that this redundancy disappears and model behavior becomes attributable to individual units of training data, resulting in what we observe as attribution decay. If this is true, then deduplication may improve attributability through decreased redundancy. Furthermore, it is then also likely that attribution decay can be observed in model classes other than diffusion models, since redundancy is a feature of the dataset rather than of the model.

Can we eliminate attribution decay by imposing some topology that localizes such redundancies to attributable units? Trivially, we could just define the entire training set to be a single unit and always correctly attribute every generated image to it, eliminating attribution decay. The interesting question is then whether finer topologies exist on which attribution decay does not occur. Our findings show that attribution decay does occur when we attempt to attribute generated images to people and to creators, so this topology would need to exist at a coarser level.

## Methods

### Defining attributability through counterfactuals

An attribution is typically defined as an explanatory cause. Therefore, we take a causal view in our analysis of attribution. Informally, we consider a sample from a generative model to be attributable to a unit of training data if, all else equal, the omission of that unit of training data would result in the generation of a different sample^17^. If no such unit exists, we say the sample is unattributable. We use the term unit here instead of single sample since that allows us to more generally define groups of images, such as those depicting a common subject or those authored by a single creator, as a single unit. Although intuitively we would like these groupings to correspond to some common characteristic, we do not impose any formal requirements. Any partitioning of the training dataset suffices.

Our causation based framework requires us to analyze counterfactual scenarios: what-if scenarios under which certain units of training data are missing. We evaluate such what-if scenarios by comparing the samples generated by two models: one which was trained on all the training data and one which was trained on all but a single unit of the training data. To adhere as closely as we can to the all else equal clause, we fix all controllable exogenous factors unrelated to the model itself such as the prompt used to guide sampling or the random Gaussian noise injected into the reverse diffusion sampling process. We refer to the sample generated by the model that was trained on all the data as the factual sample, and we refer to the sample generated by the model trained without the omitted unit of training data as a counterfactual sample. Each unit of training data induces a counterfactual sample: a sample generated by a model whose training set omits that unit. We refer to the set of all counterfactual samples of a given factual sample as its leave-one-out counterfactual universe, or simply as its counterfactual universe.

True unattributability requires that every counterfactual sample be identical to the factual sample. This is unrealistic, since there are uncontrollable exogenous factors involved in evaluating counterfactual scenarios. We therefore make the pragmatic choice to substitute identical with similar in our counterfactual analysis. Instead of checking whether every sample in the counterfactual universe of a factual sample is identical to the factual sample, we measure their similarity to the factual sample. We call the largest difference observed between a factual sample and a member of its counterfactual universe the Counterfactual Radius (CR). A sample with a low CR has low attributability, while a sample with high CR has high attributability.

### Construction of ablatable models

In conventional model training, every model parameter becomes causally entangled with every training sample. Thus, it becomes impossible to disentangle the causal structure woven between the model parameters and the individual training samples. To completely remove the causal influence of a given unit of training data, it would be necessary to train a counterfactual model from scratch.

To avoid creating this inextricable causal structure between model parameters and training data, we can instead compartmentalize model parameters into individual components that we then train independently on different subsets of training data. These components can then be combined into a single ablatable model, which as a whole will have been trained on the entire training set. We can then completely ablate the causal influence of any single unit of training data by simply removing the model components that have been trained on that unit. This idea is illustrated in Fig. 2.

Formally, we want to produce a model where given any unit of training data *s*, we are able to completely remove *s*’s influence on the model by removing some of the model’s parameters. To achieve this, we structure the model such that it is made up of components *θ*_*i*_ that have been independently trained on different splits of the training set. We also require a combining function that takes a set of trained parameters and combines them into an overall model. Crucially, the combining function should produce viable models even when model components are removed. If the combining can be done efficiently, then removing the influence of a unit of training data *s* becomes straightforward: if a factual model trained on all data is produced by combining all components *θ*_*i*_, then the counterfactual model not trained on *s* is created by combining all components that have not been trained on *s*.

We can ensure that any single unit of training data can be removed from the training set through ablation without removing any other unit of training data through careful choice of the training splits. To do this, we first pick a binary code *C* that contains at least *N* codewords of length *n*, where *N* denotes the number of units in the training set and *n* denotes the number of components we independently train. All codewords in *C* must contain the same number of 1s, and *C* can otherwise be any collection of binary strings of length *n* with a cardinality of at least *N*. Each unit of data is then assigned a unique codeword from *C*. A unit of data is included in the training split of *θ*_*i*_ if and only if the *i*th entry in its codeword is 1.

If every codeword in *C* contains a fixed number of 1s, then removing every component that was trained on a particular unit of data will guarantee that the influence of every other unit of data is retained. In other words, we can guarantee that for any other unit of data there exists a component that was not removed and was trained on that other unit of data. This is because the only way for this not to be true is if two units of data had shared a codeword, which is not the case by our construction.

If a code is constrained so that each codeword contains a fixed number of 1s, then by Sperner’s Theorem^35^ the code could contain up to *n* choose ⌈*n*/2⌉ codewords. Therefore, only *O(log(N))* components would need to be trained to allow us to produce full counterfactual universes with *N* counterfactual samples. This provides an exponential improvement in efficiency when compared to the *O(N)* models that would need to be retrained to account for all *N* counterfactual scenarios if we were not to use ablation.

### Implementation of ablatable models via diffusion ensembles

A concrete example of an ablatable model is the diffusion ensemble. Diffusion ensembles are formed by aggregating the outputs of independently trained diffusion models via arithmetic averaging. A diffusion model can be viewed as a predictor that outputs a prediction of the denoised sample upon receiving a noisy sample, so the arithmetic average of multiple predictions within an ensemble can be treated as an aggregate prediction. These predictions are then used to generate a sampling trajectory in the same way they are generated for normal diffusion models^2^, which then produces the final generated sample.

### Diffusion ensembles are viable generative models

While simple arithmetic aggregation has seen success when applied to predictive tasks, it is unclear whether this strategy is viable for generative tasks. Here we show that diffusion ensembles are indeed viable generative models. We note that image generation performance is not the focus of this study, and the purpose of this analysis is only to demonstrate the viability of diffusion ensembles. The performance can likely be further improved through hyperparameter optimization and other adjustments.

**Fig. 7:**
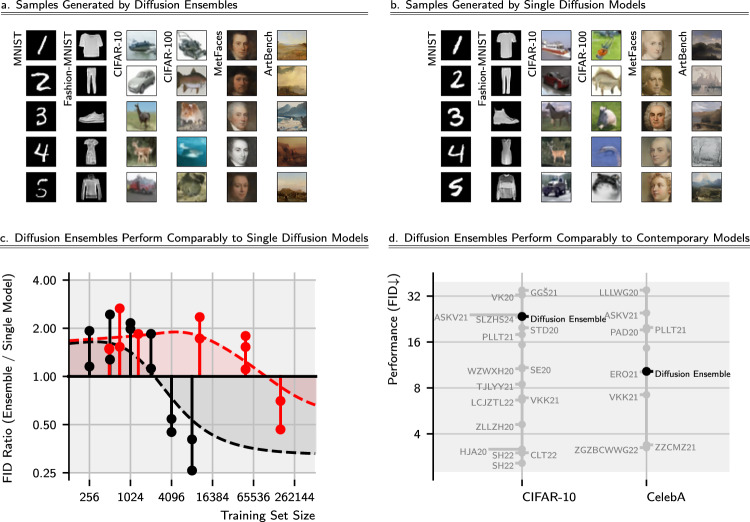
Diffusion ensembles are viable generative models. **a** Select samples generated by diffusion ensembles. 5 samples are provided for each of the 7 image distributions we trained our ensembles on. **b** Select samples generated by single diffusion models trained on the same training sets as the diffusion ensembles that produced the samples in (**a**). **c** We take the ratio of the Frechet Inception Distance (FID)^36^ attained by a diffusion ensemble against the FID attained by a single diffusion model when trained on a matching training set. We plot this ratio against the size of the training set, which reveals that ensembling tends to be more data efficient, performing relatively worse at smaller training set sizes and better at large ones (lower FID indicates improved performance). We distinguish models that were trained on grayscale and color images and plot their trends separately as they display distinct trends, though both are ultimately decreasing. Models trained on grayscale images are plotted in black, while models trained on color images are plotted in red. Dashed lines are used to indicate moving averages calculated using Gaussian Kernel Regression (details are provided in the Supplementary Information). The raw FID values are reported in Supplementary Table 4. **d** The performance of diffusion ensembles benchmarked against those of various existing contemporary image generation methods reported in the literature^2,37–55^ as measured by the FID. We compare image generators trained on CIFAR-10 and CelebA as an abundance of FID measurements on these datasets exist in the literature. The FID marked with the Diffusion Ensemble label is the best FID achieved by a diffusion ensemble trained on the given image distribution.

Qualitatively, we provide a selection of samples generated by these diffusion ensembles in Fig. 7a for visual evaluation. As reference, we trained and evaluated 24 single diffusion models on the exact same datasets we used to train the diffusion ensembles. Diffusion models when not ensembled are known to achieve state-of-the-art performance in image generation^2^, so this provides a reference for how ensembling improves or degrades performance, all else being equal. Samples generated by these single diffusion models are presented in Fig. 7b. We observe that the samples are of comparable quality, with the exception of the samples generated by ensembles trained on MetFaces, which are noticeably blurrier. We note that MetFaces contains far fewer images than the other image distributions displayed here, and that the blurriness of images generated by ensembles trained on small datasets is a general phenomenon that can also be observed in Figs. 3 and 5. This increased blurriness disappears when more training data is available.

We quantified the performance of these ensembles with the Frechet Inception Distance (FID)^36^, a popular metric for benchmarking generative models in the literature that measures how distinct generated image distributions are from desired natural image distributions. The relative performances of the diffusion ensembles compared with those of the single diffusion models are reported in Fig. 7c.

We observe that their performances are comparable, rarely differing by a factor much greater than 2 in either direction. We note that the relative performance of diffusion ensembles against diffusion models tends to improve as the amount of training data increases. This suggests that diffusion ensembles are more data efficient than diffusion models, which may be of independent interest. We note this is also in part a reflection of the previous observation that ensembles trained on small datasets exhibit blurriness in the small dataset regime where elevated FID can be observed.

We also compared the performances of our diffusion ensembles trained on CIFAR-10 and CelebA against the performances of contemporary generative models trained on those image distributions reported in the literature^2,37–55^. We find that the diffusion ensemble is within the range of performances exhibited by contemporary techniques. The FIDs are reported in Fig. 7d.

### Metrics used to quantify image similarity

The CR of a generated factual image is the largest distance to an image in its counterfactual universe. We use the Euclidean distance, a geometric metric, as our main measure of image distance. Euclidean distance is calculated by treating each image as a vector of pixel channel intensities and inheriting the Euclidean metric from the real coordinate space. We note that this distance can vary greatly depending on image dimension, so we first scale all images to 256 × 256 (with nearest neighbor sampling) and express all greyscale images in RGB space by duplicating the channels first before computing the Euclidean distance to ensure distances are comparable across images from different image distributions. We also repeated our analysis using 3 alternative metrics to quantify image similarity to ensure our findings are robust to the choice of image similarity metric:

- OpenCLIP, a semantic metric
- LPIPS, a perceptual metric
- DINOv2, another semantic metric

OpenCLIP embeddings were computed by using the ViT-H-14-378-quickgelu model pretrained on dfn5b. LPIPS was run in VGG mode. DINOv2 embeddings were computed with the ViT-L/14 model with registers. For both OpenCLIP and DINOv2, the distances were calculated by first computing the embeddings and then computing the cosine distance between the embeddings. In our main results we report the results we attain using the Euclidean distance (geometric) and OpenCLIP (semantic). The results we attain using the other metrics are reported in the Supplementary Figs..

### Image similarity based attribution

For our experiments involving FAR calculations, we used image similarity based attribution. To attribute generated images to training data using image similarity, we first selected an image similarity metric (Euclidean, OpenCLIP, LPIPS, or DINOv2). Using the metric of choice, we computed the distance between the generated image to every image within the training set. While training on select image distributions, we used horizontal mirroring as a data augmentation strategy, which is documented in Supplementary Table 1 in the Supplementary Information. In these cases, we additionally computed the distance between the generated image and horizontally flipped versions of the training images. The final distance was then taken to be the minimum of the flipped and unflipped distance. Finally we take the training image that is the closest to the generated image to be the attributed image. If a unit of training data consists of more than one image, we return the unit that contains the closest training image.

### Calculating the false attribution rate

The FAR is calculated from two probabilities: the probability that the attribution of a generated sample remains unchanged upon omitting the attributed sample, which we denote as *p*_*1*_, and the probability that it remains unchanged upon removing a randomly selected unattributed sample, which we denote as *p*_*2*_. *p*_*2*_ is used to adjust for uncontrolled exogenous factors.

There are two events that we need to consider. Let *A* denote the event that the attribution was correct. Let *B* denote the event that some uncontrolled exogenous factors caused the attribution to change under a counterfactual scenario. We think of *p*_*2*_ as an estimate of *P(not B)*, or 1 - *P(B)*.

Our use of the FAR is premised on the idea that *A* cannot be true if the attribution remains unchanged upon omitting the attributed sample. Therefore, *p*_*1*_ = *P(not A ∩ not B)*. If we assume *A* and *B* are roughly independent, then *P(not A ∩ not B)* ≈ *P(not A)P(not B)* = *p*_*2*_*P(not A)*. Therefore, the probability of an incorrect attribution, *P(not A)*, is approximated by *p*_*1*_/*p*_*2*_, which we define to be the False Attribution Rate. Since this is a probability, we clip this value to a range between 0 and 1.

To construct 1-*x* confidence intervals for FARs, we first constructed Clopper-Pearson intervals of confidence 1-(*x*/2) for *p*_*1*_ and *p*_*2*_. The final confidence interval is then given as the interval containing all values that can be obtained given that both *p*_*1*_ and *p*_*2*_ fall within their confidence intervals.

### Statistics

Statistical tests and *p*-values are reported in the Results section where relevant. We summarize the statistical tests we performed in this section.

To determine if the relationships presented in Fig. 4a, d are statistically significant, we performed linear regression between the logs of the training set sizes and mean CRs. The one-sided Wald test was then used to confirm that the slope of these relations are negative with significance. We attain *p* < 10^−5^ for both geometric and semantic CRs with *N* = 24 in both cases.

The one-sided Mann-Whitney U test was used to establish that CR distributions generated from ensembles and models trained on more data are significantly lower than CR distributions generated from models trained on less data. We list the tests we performed, the number of samples used in them, and the *p*-values we obtained from them in Supplementary Table 5 in the Supplementary Information.

## Supplementary information

Supplementary Information

Transparent Peer Review file

## Data Availability

The processed counterfactual universe distance measurements have been deposited in Zenodo under accession code 10.5281/zenodo.20014931. ^56^ A subset of the raw generated image data has been deposited in a GitHub repository under accession code 10.5281/zenodo.20965172. ^57^ The remaining raw image data generated during the study are not publicly available due to the volume of raw image data generated for this study, but indefinite access can be obtained from the corresponding author on request provided a file sharing solution. Requests for materials or other correspondence may be addressed to either author at {zhengdai,gifford}@mit.edu.

## Code availability

The code and data supporting the analyses in the current study are accessible through our website at https://zheng-dai.github.io/AblationBasedCounterfactuals/. Scripts for replicating our pipeline are publicly available in our Github repository under accession code 10.5281/zenodo.20965172. ^57^
