# Supplementary material for: Outputs of generative diffusion models are often unattributable
Source: Nat Commun. 2026 Aug 18;17:6974. doi: 10.1038/s41467-026-75667-5 (PMC13487234; doi:10.1038/s41467-026-75667-5)
Supplement: Supplementary file 1 [file 41467_2026_75667_MOESM1_ESM.pdf]

Outputs of Generative Diffusion Models are Often Unattributable

Supplementary Information

- Supplementary Notes
- Supplementary Figures (1-7)
- Supplementary Tables (1-6)

Supplementary Notes

Training Data Preparation

For our main study, we trained 24 diffusion ensembles on subsets derived from 7 different image datasets with varying characteristics. To avoid ambiguity with the actual training sets of the models which are derived from these image datasets, we shall henceforth refer to these image datasets as *image distributions*. The image distributions are as follows:

- MNIST (Dataset Card: https://huggingface.co/datasets/ylecun/mnist) consists of grayscale images of handwritten digits at low resolution (28-by-28).
- Fashion-MNIST (Dataset Card: https://huggingface.co/datasets/zalando-datasets/fashion_mnist) is a drop-in replacement for MNIST consisting of grayscale images of fashion items at low resolution (28-by-28).
- CIFAR-10 (Dataset Card: https://huggingface.co/datasets/uoft-cs/cifar10) consists of low resolution (32-by-32) images depicting a variety of subjects from 10 object classes.
- CIFAR-100 (Dataset Card: https://huggingface.co/datasets/uoft-cs/cifar100) is similar to CIFAR-10 except it contains 100 object classes.
- CelebA (Dataset Card: https://huggingface.co/datasets/flwrlabs/celeba) consists of images depicting the faces of celebrities. Image dimensions vary.
- MetFaces (Repository: https://github.com/NVlabs/metfaces-dataset) consists of portrait paintings. Image dimensions vary.
- ArtBench (Repository: https://github.com/liaopeiyuan/artbench) consists of artwork at a 256-by-256 resolution.

We applied the following processing to these image distributions: images from MNIST and Fashion-MNIST were padded to be 32-by-32 by adding a two-pixel wide black border. CelebA was center cropped to be 128-by-128. MetFaces was scaled to 256-by-256 via bilinear interpolation.

Data was prepared for latent diffusion for images from the CelebA, MetFaces, and ArtBench image distributions, while pixel space diffusers were trained for the other image distributions. The Stable-Diffusion-v1-5 autoencoder[4] was used to embed the CelebA, MetFaces, and ArtBench images to a latent space with dimension 4x32x32. In the case of CelebA, images

were upscaled to 256-by-256 via bilinear interpolation before embedding. Images in CelebA were additionally embedded to a different latent space using a "basic autoencoder" that we trained in-house.

We also produced a binarized version of the MNIST image distribution by taking each image and rounding each pixel value to 0 or 1 with a threshold of 0.5 and removing any duplicate images that this produced. In total, this gives us a total of 9 processed image distributions, since we processed CelebA using two different autoencoders and we processed MNIST both normally and with binarization. We use CelebA (SD) and CelebA (BA) to disambiguate between the CelebA image distribution that was processed using the Stable Diffusion Autoencoder and Basic Autoencoder respectively. We use MNIST (Binarized) to specify the processed MNIST image distribution that was binarized. These 9 processed image distributions and their properties are summarized in Supplementary Table 1.

Construction of a basic autoencoder

Although we primarily relied on the Stable-Diffusion-v1-5 autoencoder[4] as an off-the-shelf solution for latent diffusion, we also trained a basic autoencoder in-house on the test split of the CelebA image distribution, which is disjoint from the data we used to train diffusion models and ensembles. This was done to ensure that our observations were robust to autoencoder selection.

The basic autoencoder maps between images with shape 128-by-128 and latent embeddings of shape 4x32x32. The encoder is made of 3 convolutions (channel widths going from 3 to 64 to 64 to 4), downscaling by a factor of 2 for the first two steps, and with kernel sizes of 7x7, 7x7, and 5x5 respectively. The decoder is made of 2 transposed convolutions that each upscale by a factor of 2 followed by 2 convolutions. Kernel sizes are 7x7, 7x7, 5x5, and 3x3 respectively. A ReLU follows each convolution, and after the second ReLU in the encoder a BatchNorm2d is used.

This autoencoder was trained on the test split of the CelebA image distribution. The initial objective was to minimize mean squared error between the original image and the image after it has been passed through the autoencoder. This phase lasted for 10 epochs. Adam was used for gradient descent with default parameters, and a batch size of 64 was used. After this, the loss was adjusted to incorporate patches to improve local detail. To calculate this loss, the image was divided into 8x8, 4x4, 2x2, and 1x1 patches. The mean squared loss was calculated for each patch at each patch size, and the maximum was taken over all patches at each patch size. The losses were then scaled by 4, 8, 16, and 32 respectively and added to the default mean squared error loss. Training continued for an additional 574 epochs with this adjusted loss.

Model training

We derived smaller subsets from the nine processed image distributions enumerated in Supplementary Table 1. For MNIST and Fashion-MNIST, we randomly selected subsets of size 256, 512, 1024, 2048, 4096, and 8192 from their test splits. For CIFAR-10 and CIFAR-100, we randomly selected subsets of size 500 and 50000 from their training splits. For these 16 training sets, we consider each image to be its own unit of data. For CelebA (both SD and BA variants), we randomly selected subsets of size 712, 10627, and 162770 from its training split, representing 32, 512, and 8192 celebrities respectively. For these 6 training sets we consider a set of images depicting a single celebrity to be a single unit of data. For MetFaces we used the entire image distribution, and for ArtBench we used the entire training split. The datasets contain 1336 and

50000 samples respectively, representing the work of 744 and 2108 artists respectively. For these 2 training sets we consider a set of images created by a single artist to be a single unit of data.

This accounts for the training sets used for the 24 ensembles that we used to obtain our main results. These ensembles are documented in Supplementary Table 2. Additional ensembles trained for this study are discussed in later sections, and are documented in Supplementary Table 3. We will henceforth use the identifiers presented in these tables to identify ensembles and training sets in question.

Models were trained by predicting denoised images using the scheme proposed in "Denoising diffusion probabilistic models".[2] 1000 possible noise levels were used for training, although sampling was performed with only 50 denoising steps. Random horizontal flipping was used as a data augmentation strategy for certain image distributions (please consult Supplementary Table 1 for a full list). Adam was used for gradient descent with default PyTorch parameters, a learning rate of 0.0001, and a batch size of 128. Two variants of the U-Net were used based on the Hugging Face Diffusers implementation. Each U-net consisted of 4 down and upblocks, with an attention layer on the second down and third upblock. The channel sizes on each layer of the U-Net was (128, 256, 512, 512) for the smaller U-Net and (192, 384, 768, 768) for the larger U-Net. Supplementary Table 2 and 3 document the architectures used for each ensemble. A dropout of 0.1 was applied to certain models, which is also documented in Supplementary Table 2 and 3. During training, images were normalized by subtracting a mean value and then dividing by a standard deviation value specific to image distributions. The mean and standard deviations used for this normalization purpose are reported in Supplementary Table 1.

Details on FID calculation

We used the FID to measure generative model performance. The mean and covariances of the embeddings of the generated distributions were computed using 102400 samples. Bessel's correction was applied for covariance calculation (i.e. normalization by $N-1$ instead of N, where N is the number of samples). The image distributions our training sets were derived from consist of both a split that intersects with our training sets and a split that is disjoint from it (see Supplementary Table 1 for which split was used for training). The FID attained when using the former and the latter split as the reference dataset are both reported in Supplementary Table 4. The FIDs reported in Figure 7 are those attained when using the disjoint reference distributions, with the exception of MetFaces, where a disjoint split does not exist.

Deriving a relation between CR and Training Set Size

We quantified the strength of attribution decay by fitting a power law between the mean CR of ensembles and the size of the datasets they were trained on. Fitting was achieved through an ordinary least squares fit between the logarithms of the two sets of values. This produced negative relationships with R^2 values of 0.85, 0.59, 0.29, and 0.15 for CRs measured with the Euclidean distance, OpenCLIP, LPIPS, and DINOv2 respectively. Note that these R^2 values are those of the ordinary least squares fit performed on the logarithms of the values. Using the one-sided Wald test $(N=24)$ yields p-values less than 10^{-10}, 10^{-5}, 0.0032, and 0.031 respectively for these fits. The first two relationships are presented in Figure 4, and the rest are presented in Supplementary Fig. 1.

To compute the mean CR for each ensemble, 100 samples were generated from each ensemble, with the exception of CIFAR10_50000 and CIFAR100_50000 (see Supplementary

Table 3), where only 25 samples were generated due to the size of the counterfactual universes of these samples. CRs were calculated by generating each counterfactual sample in the counterfactual universe of a factual sample and then finding the counterfactual sample with the largest distance from the factual sample. Distances were measured using all 4 similarity metrics discussed in the Methods section. The mean CR of an ensemble is then given by the arithmetic average across all samples generated by that ensemble.

Image distribution specific counterfactual radius (CR) adjustments

Although the training set size alone is a good predictor of CR, some image distribution dependent idiosyncrasies can be observed (see the first row of Supplementary Fig. 1). To see what would happen if we controlled for these effects, we performed multiple linear regression, where the log CR was used as the target variable and the explanatory variables consisted of both the log training set size and a set of 7 indicator variables that indicate which image distribution the training set was derived from. Applying these adjustments increases the R^2 to above 0.9 for all 4 types of CR. The adjusted relation is shown in Supplementary Fig. 1, where we also show the adjustment factors we derived. From these values we can see that ensembles trained on image distributions with high semantic variability like CIFAR-10 and CIFAR-100 tend to have high CR when measured using semantic distances (OpenCLIP and DINOv2).

Analyzing drops in CR conditioned on unit type

To analyze attribution decay when conditioned on unit types, we categorized our ensembles based on what was considered a single unit of data as follows:

- Image: MNIST_256, MNIST_512, MNIST_1024, MNIST_2048, MNIST_4098, MNIST_8192, FASHION_256, FASHION_512, FASHION_1024, FASHION_2048, FASHION_4098, FASHION_8192, CIFAR10_500, CIFAR10_50000, CIFAR100_500, and CIFAR100_50000
- Person: CELEBA_BA_32, CELEBA_BA_512, CELEBA_BA_8192, CELEBA_SD_32, CELEBA_SD_512, CELEBA_SD_8192
- Artist: METFACES, ARTBENCH

We then took the ensembles that were trained on the least amount of data for each group:

- MNIST_256, FASHION_256
- CELEBA_BA_32, CELEBA_SD_32
- METFACES

And combined the CR distributions they generate, yielding 200, 200, and 100 samples respectively. We then took the ensembles that were trained on the most amount of data for each group:

- CIFAR10_50000, CIFAR100_50000
- CELEBA_BA_8192, CELEBA_SD_8192
- ARTBENCH

And combined the CR distributions they generate, yielding 50, 200, and 100 samples respectively. We then checked whether the former distributions are significantly larger than the latter ones using the one-sided Mann-Whitney U test. For both geometric distance as measured by the Euclidean distance and semantic distance as measured by OpenCLIP, we achieve significance with $p<10^{-4}$.

A more detailed breakdown with all four image similarity metrics is presented in Supplementary Fig. 2, where every CR distribution by ensemble is shown, grouped by the image distribution their training sets were derived from (although we group METFACES and ARTBENCH together given that only one training set was derived from each image distribution). This is a strictly finer breakdown than by unit type. We report p-values attained when comparing the CR distribution obtained from the ensemble trained on the smallest training set to that of the CR distribution obtained from the ensemble trained on the largest training set within each group in Supplementary Table 5 (first 7 rows). Additional statistical details like sample size are also reported in Supplementary Table 5. The p-values are obtained using the one-sided Mann-Whitney U test, with the alternative hypothesis that the former distribution is larger than the latter. We find that $p<0.0012$ across all 7 groups for all 4 image similarity metrics used to measure CR.

Analyzing attribution decay in ensembles that are class conditioned

We trained 12 class conditioned ensembles on MNIST and Fashion-MNIST (documented in Supplementary Table 3 as MNIST_CLASS_<training set size> and FASHION_CLASS_<training set size>). Class conditioning was achieved by learning an embedding for each class that gets added to the timestep embedding, which then guides the class conditioned sampling. MNIST and Fashion-MNIST natively support 10 distinct classes each, which were used as class inputs. 10 samples and their counterfactual universes were generated for each class, resulting in a total of 100 samples and their counterfactual universes for each ensemble. The distributions of the CRs of these samples are shown in Figure 4 and Supplementary Fig. 5.

We observe a significant drop in CR distributions when moving from training set sizes of 256 to 8192 via the one-sided Mann-Whitney U test for both ensembles trained on MNIST and Fashion-MNIST ($p<10^{-11}$ for all image similarity metrics, a full breakdown presented on rows 8 and 9 of Supplementary Table 5).

Analyzing attribution decay in ensembles that are text prompt conditioned

We trained text conditioned ensembles on CelebA (documented in Supplementary Table 3 as CELEBA_TEXT_<training set size>). In text conditioned models, text is first encoded and embedded using the Stable-Diffusion-v1-5 pipeline. The embedding is then consumed via a cross-attention mechanism described in “High-resolution image synthesis with latent diffusion models”[4], which then allows for text guided sampling. CelebA supports a vector of binary flags that indicate features present in each picture, which we use to derive text prompts. Each text prompt starts with “Face of a man” or “Face of a woman” based on the gender flag, followed by a comma separated list of features such as “eyeglasses” or “wearing hat” based on the other flags, which are appended in a randomly permuted order. To generate samples, we created 50 prompts that are disjoint from what was observed in the training set, but are similarly structured. We also used 50 prompts that were in the training set. A few example prompts are:

- “Face of a man, bags under eyes, big nose” (from the training set)
- “Face of a woman, narrow eyes, mouth slightly open, black hair” (from the training set)
- “Face of a woman, gray hair, wearing necklace” (disjoint from the training set)
- “Face of a man, bangs, wearing necklace, wearing necktie” (disjoint from the training set)
- “Face of a man, bags under eyes, big nose” (disjoint from the training set)

Together, these 100 prompts were used to generate 100 samples and their counterfactual universes for each ensemble. The distributions of the CRs of these samples are shown in Figure 4 and Supplementary Fig. 5.

We observe a significant drop in CR distributions when moving from a training set size of 712 to 162770 via the one-sided Mann-Whitney U test ($p<10^{-19}$ for all image similarity metrics, a full breakdown is presented on row 10 of Supplementary Table 5).

Analyzing attribution decay on ensembles that are trained for the same number of epochs

During training, we would generally vary the number of training epochs so ensembles trained on the same image distribution get trained for roughly the same number of iterations (see Supplementary Table 2 and 3 for the number of epochs each ensemble is trained for). This means that samples in smaller datasets were seen less often. To control for this factor, we trained an ensemble analogous to CELEBA_SD_512 that was trained for roughly 4 times less epochs and an ensemble analogous to CELEBA_SD_8192 that was trained for roughly 4 times more epochs. The resulting ensembles were trained for an equal number of epochs. They are listed as CELEBA_EPOCH_512 and CELEBA_EPOCH_8192 in Supplementary Table 3.

We observe a significant drop in CR distributions when moving from a training set size of 10627 to 162770 via the one-sided Mann-Whitney U test ($p<0.002$ for all image similarity metrics, a full breakdown is presented on row 11 of Supplementary Table 5).

Analyzing attribution decay with fixed proportion units

To create a “Fixed Proportion” experimental setting, we trained a set of ensembles on subsets of the MNIST and Fashion-MNIST image distributions where we held the number of units constant at 96 while varying training set sizes from 256 to 8192 samples. These ensembles are documented as MNIST_96_<training set size> and FASHION_96_<training set size> in Supplementary Table 3.

The units were obtained by randomly partitioning the training split of the MNIST and Fashion-MNIST image distribution into 96 evenly sized units. The resulting ensembles have the property that the proportion of data that gets removed in each counterfactual scenario remains roughly constant regardless of training set size, which allows us to determine if attribution decay is a function of training data size or if it is a function of the proportion of data removed. 100 samples and their counterfactual universes were generated for each ensemble. The distributions of the CRs of these samples are shown in Figure 4 and Supplementary Fig. 5.

We observe a significant drop in CR distributions when moving from training set sizes of 256 to 8192 via the one-sided Mann-Whitney U test for both ensembles trained on MNIST and Fashion-MNIST ($p<10^{-3}$ for all image similarity metrics, a full breakdown is presented on rows 12 and 14 of Supplementary Table 5).

Analyzing attribution decay without using ablation

To ensure that our results are not an artifact of our ablation based methodology, we reproduced our results on a smaller scale through a more conventional retraining approach, where the influence of a unit of training data was removed by retraining models from scratch on datasets without the unit of training data in question. We took subsets of size 256 and 1024 from the MNIST and Fashion-MNIST image distributions and trained diffusion models on those subsets and every leave-one-out variant (i.e. a subset with one image missing). A total of 2564 models were trained. The models trained on the full datasets were used to generate factual samples, while the models trained on leave-one-out variants were used to generate counterfactual samples. 100 samples and their counterfactual universes were generated for each dataset. The distributions of the CRs of these samples are shown in Figure 4 and Supplementary Fig. 5.

We observe a significant drop in CR distributions when moving from training set sizes of 256 to 1024 via the one-sided Mann-Whitney U test for both ensembles trained on MNIST and Fashion-MNIST ($p<10^{-3}$ for all image similarity metrics, with full statistical details presented on rows 13 and 15 of Supplementary Table 5).

Creating an ensemble to generate binarized MNIST images

To see if we can produce zero CR samples, we trained an ensemble to generate binarized MNIST images (documented as MNIST_BINARIZED in Supplementary Table 3). This ensemble was trained on a binarized version of the MNIST image distribution (denoted MNIST (Binarized) in Supplementary Table 1).

The diffusion process still operates on a continuous space, so a final binarization step is applied at the end of the diffusion process to round pixel values to either 0 or 1 using 0.5 as a threshold.

Alternative summaries of counterfactual universes

We plot alternative summaries of the counterfactual universe in Supplementary Fig. 6 to provide more insight into the structure of these universes outside of the CR. The CR is the 100th percentile of the distances between the counterfactual samples and the factual sample. Therefore, for our alternative summaries we add the 75th, 50th, and 25th percentiles, as well as the mean of the distances. Similar to our analysis in Supplementary Fig. 1, we average the summary statistics for each diffusion ensemble and plot the relation between these statistics and the training set size.

We find that while a negative relation between these alternative summary statistics and training set size is preserved, the strength of the relation tends to diminish as we move into the lower percentiles.

Comparing counterfactual attributability to similarity based retrievability

A commonly used proxy for the attributability of a generated image is the existence of samples in the training data whose similarity to the generated image is under some threshold.[32, 33] We will refer to these methods as retrievability based methods, and we can quantify the similarity based retrievability of a generated sample as the minimum distance between it and a sample in the training data. To better position attributability in the context of these existing measures, we compare attributability as defined by CRs with retrievability.

To compute retrievability, we first selected an image similarity metric (Euclidean distance, LPIPS, OpenCLIP, or DINOv2). Using the metric of choice, we computed the distances between the generated images and every image within the training set. For certain image distributions, we used horizontal mirroring as a data augmentation strategy during training, which is documented

in Supplementary Table 1. In these cases, we additionally computed the distances between the generated images and horizontally flipped versions of the training images. The minimum distance measured is then multiplied by negative one to obtain the retrievability of the generated sample. The negation means that more retrievable samples have larger retrievability values, similar to attributability.

Once a set of generated samples were assigned attributability and retrievability values, we used the Kendall rank correlation coefficient to measure the concordance between the values. We chose this measure because the Kendall rank correlation coefficient can be interpreted as a measure of agreement between two binary classifiers (in this case whether a sample is attributable and whether a sample is retrievable) when the classification thresholds are given by a randomly selected benchmark sample.

We measured the correlations over the 2250 samples we generated from the 24 ensembles listed in Supplementary Table 2. The correlations were measured individually for each of the 7 image distributions to reduce confounding factors. The arithmetic average of these correlations is reported in Supplementary Table 6. Attribution and retrievability are most concordant when the CR is measured using LPIPS and when retrievability is measured with the Euclidean distance, where the correlation is 0.157. This is higher than the concordance between some of the different retrievability measures, for example the correlation between retrievability when measured with the Euclidean distance and retrievability when measured with OpenCLIP is only 0.092.

We emphasize that concordance here should not be conflated as a measure of performance, but rather as a way of contextualizing CR based attributability in the landscape of related measures.

Calculating moving averages

Figure 6 and Figure 7 both feature a moving average that helps visualize trends in the data. These were computed by performing a Gaussian kernel regression in (natural) log space with a standard deviation of 1.

Calculating confidence intervals of medians

Figure 4 features notched box plots which indicate the 95% confidence intervals of the median. This interval surrounds the median symmetrically, and extends 1.57 times the interquartile range divided by the square root of the number of samples in both directions.

Computing Resources

This study was performed on 8 Titan RTX GPUs (24190MiB), 8 GeForce GTX 1080 Ti GPUs (11264MiB), and 10 GeForce RTX 2080 Ti GPUs (11264MiB) running for approximately 10^3 hours each on average.

Supplementary Figures

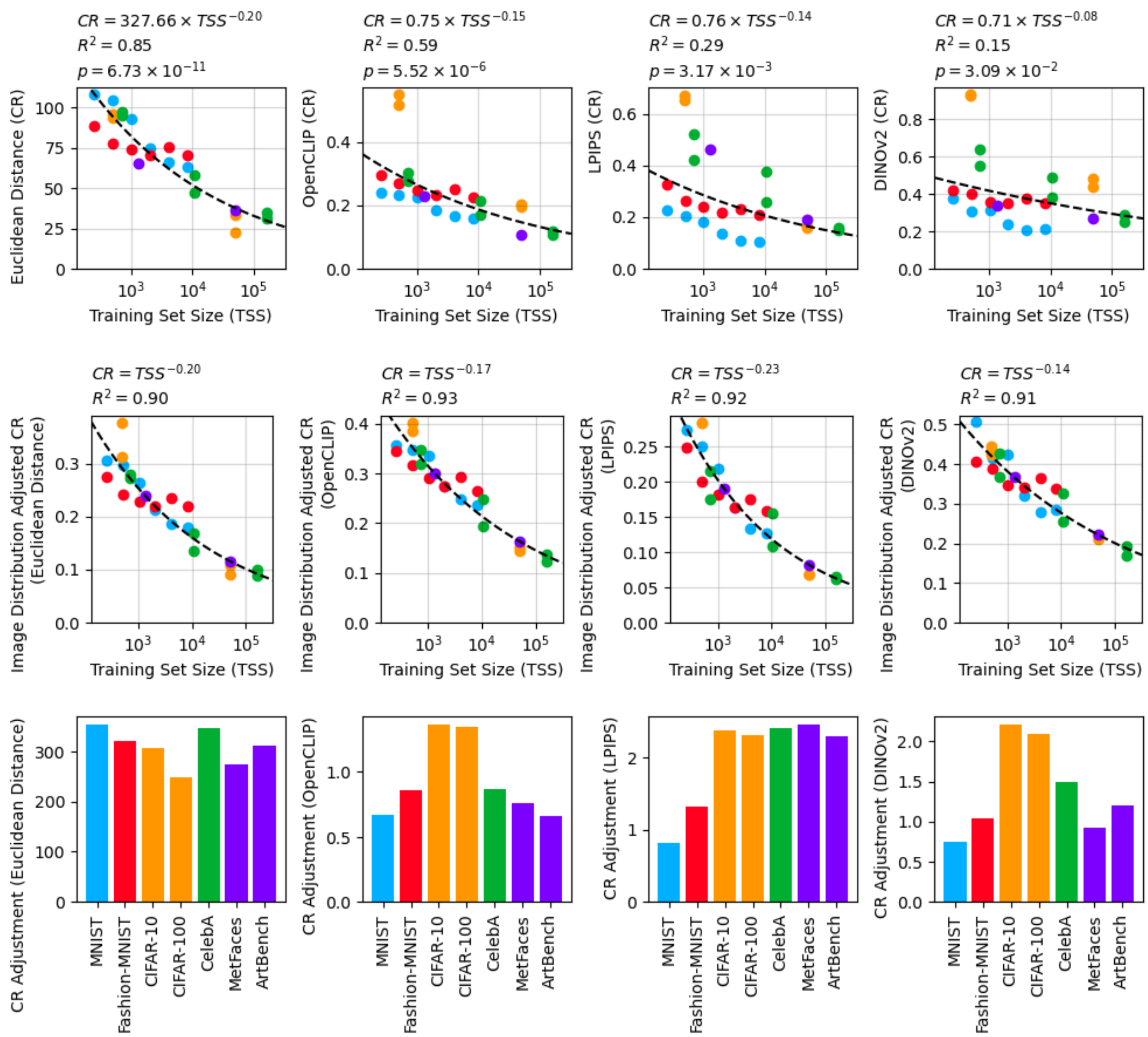

Supplementary Fig. 1. Attribution decay

Top row: Extended version of the scatter plots in Figure 4 showing the relation between training set size and CR measured in Euclidean Distance, OpenCLIP, LPIPS, and DINOv2. The R^2 is also reported along with p-values for the one-sided Wald test with the alternative hypothesis that the slope is negative. The colors of the points are congruous with those in Supplementary Fig. 2 (blue for ensembles trained on MNIST, red for Fashion-MNIST, yellow for CIFAR-10 and CIFAR-100, green for CelebA, and purple for MetFaces and Artbench). The power law is shown above the figure, where TSS stands for "training set size", and is plotted as a dashed line.
Middle row: Power laws obtained when CRs are adjusted for image distribution.
Bottom row: The adjustment factors extracted through multiple linear regression. Multiplying the adjusted CRs plotted in the middle row by these factors yields the true CRs in the top row.

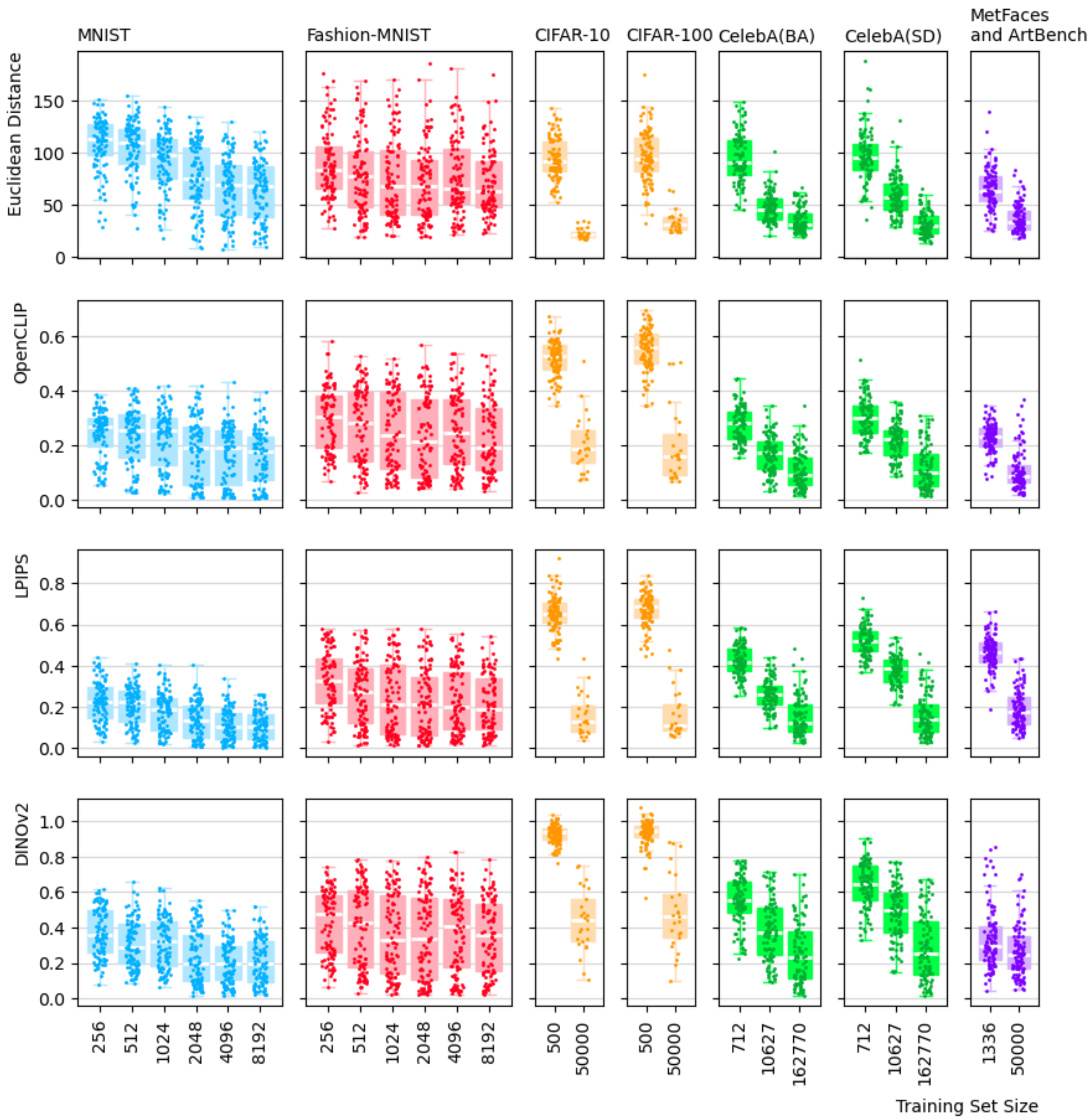

Supplementary Fig. 2. Counterfactual Radius distributions

The full distributions of the CRs that were averaged in Supplementary Fig. 1 across image distributions (horizontal) and distance metrics (vertical). The strip plots show the full CR distribution while the box plots span the first to the third quartile, with whiskers extending either 1.5 times further or to the furthest point. The median is marked by a white line.

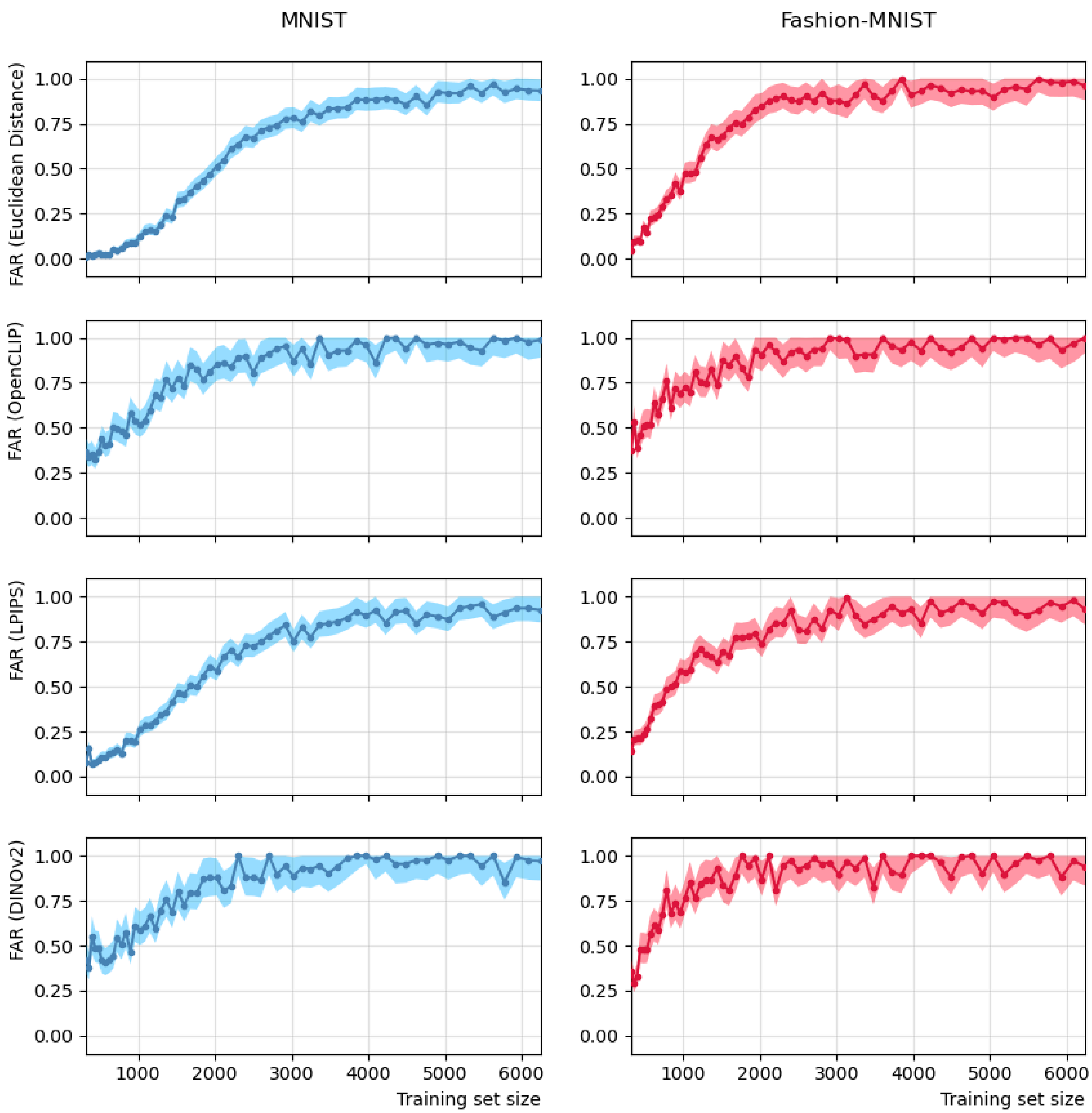

Supplementary Fig. 3. False Attribution Rate trajectories for MNIST and Fashion-MNIST
Extended figures of the trajectories shown in Figure 6, showing how the FAR monotonically increases for various image similarity based attribution methods when training set sizes are increased from 324 to 6241. Each curve is made of 62 points. Ensembles are trained on both MNIST (left) and Fashion-MNIST (right). For attribution purposes, image similarity was measured with Euclidean distance, OpenCLIP, LPIPS, and DINOv2. The shaded bands provide the Clopper-Pearson 95% confidence intervals *(N=1024)*. Details regarding the calculation of the Clopper-Pearson confidence interval are given in the Methods section.

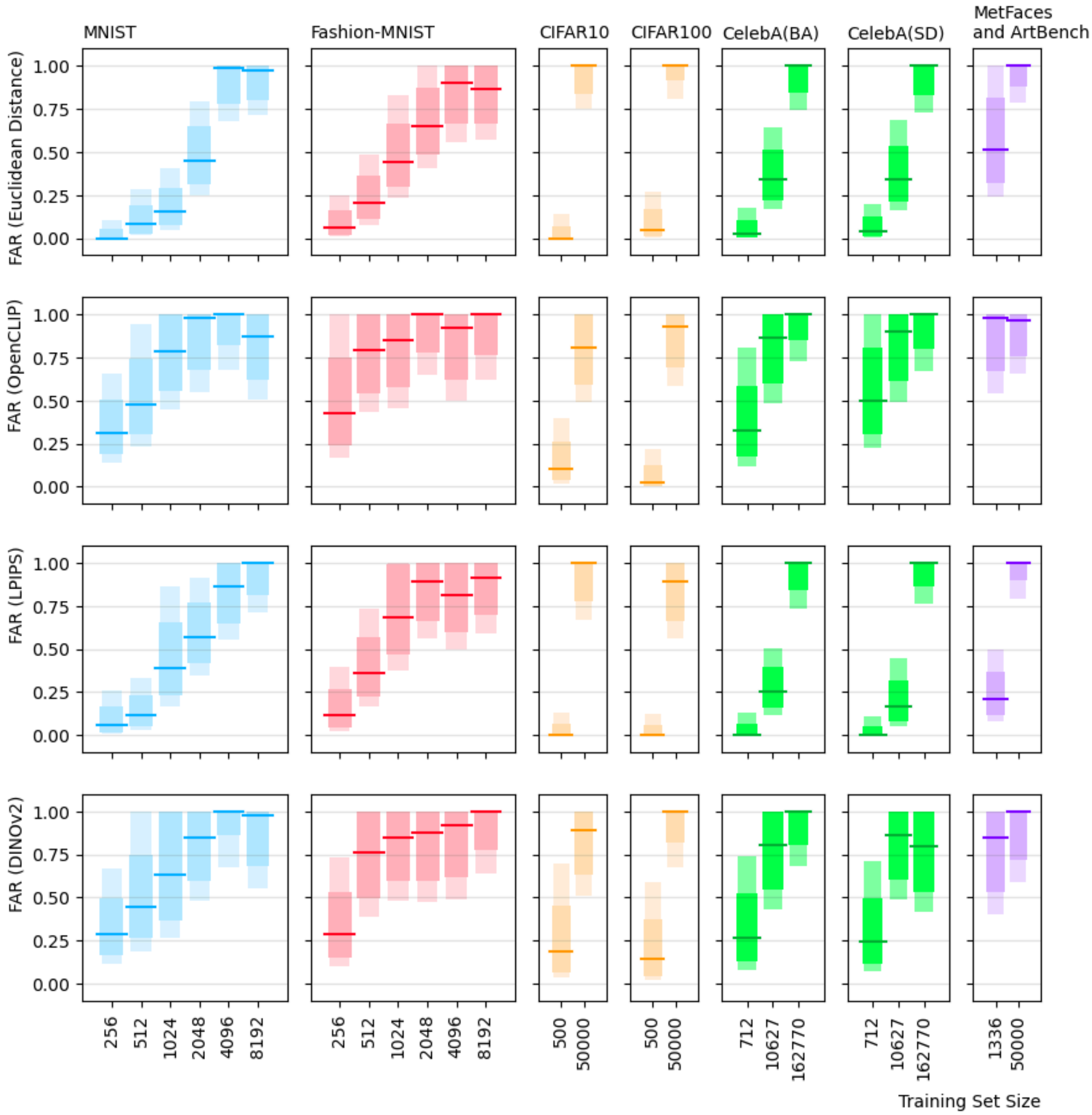

Supplementary Fig. 4. False Attribution Rates for ensembles across image distributions

Extended figure of FARs plotted in Figure 6 organized by image distribution and including additional image similarity attribution methods (Euclidean distance, OpenCLIP, LPIPS, and DINOv2). The horizontal bar marks the FAR point estimate, the darker region marks the Clopper-Pearson 66.6% confidence interval, and the lighter region marks the Clopper-Pearson 95% confidence interval *(N=100)*. Details regarding the calculation of the Clopper-Pearson confidence interval are given in the Methods section.

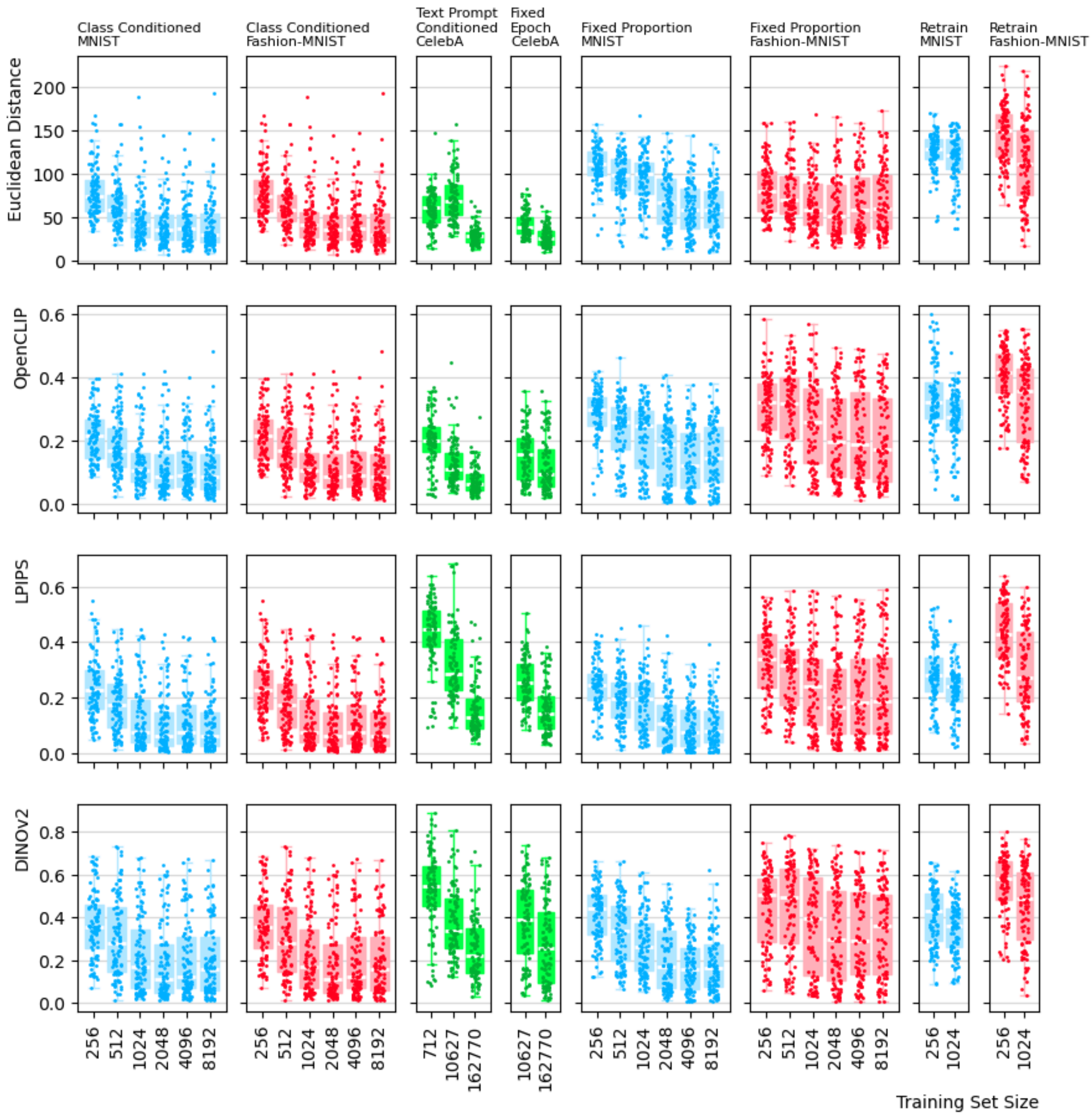

Supplementary Fig. 5. Counterfactual Radius distributions for additional experimental conditions

Extended version of Figure 4, with CRs measured in Euclidean distance, OpenCLIP, LPIPS, and DINOv2. In addition, CRs from class conditioned ensembles trained on Fashion-MNIST, CRs from ensembles where counterfactual scenarios remove a fixed proportion of data trained on Fashion-MNIST, and retraining based CRs from models that were trained on Fashion-MNIST are included. The strip plots give the CR distribution while the box plots span the first to the third quartile, with whiskers extending either 1.5 times further or to the furthest point. The median is marked by a white line.

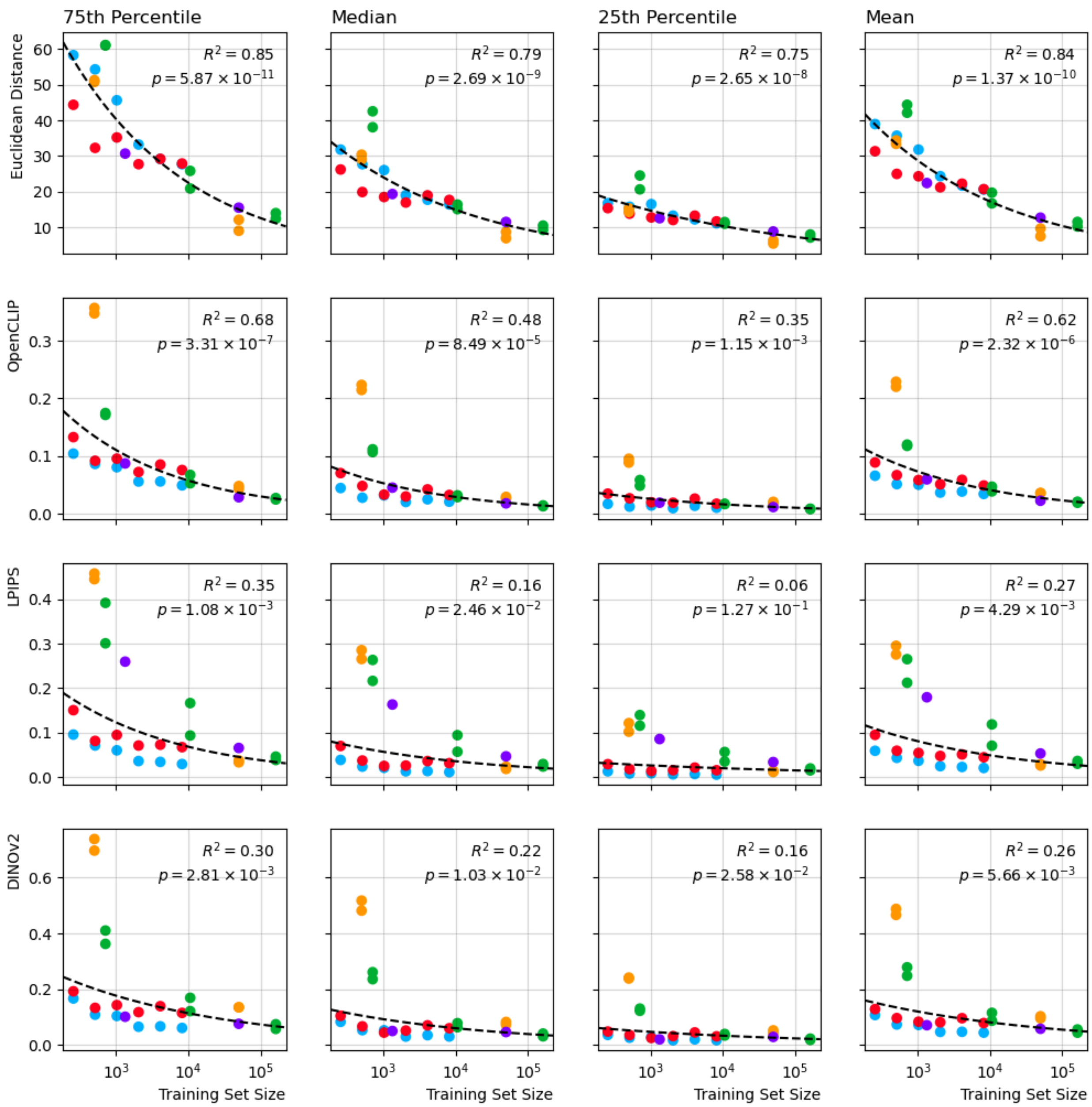

Supplementary Fig. 6. Alternative summaries of counterfactual universes and their relation to training set size

We show the relations between training set size and alternative summaries of counterfactual universes. The alternative summaries are the 75th percentile of the distances between the factual and counterfactual samples, the 50th percentile (median) of the distances, the 25th percentile of the distances, and the mean of the distances. The summary metrics are averaged over each diffusion ensemble as was done in Supplementary Fig. 1. The R^2 is also reported along with p-values for the one-sided Wald test with the alternative hypothesis that the slope is negative.

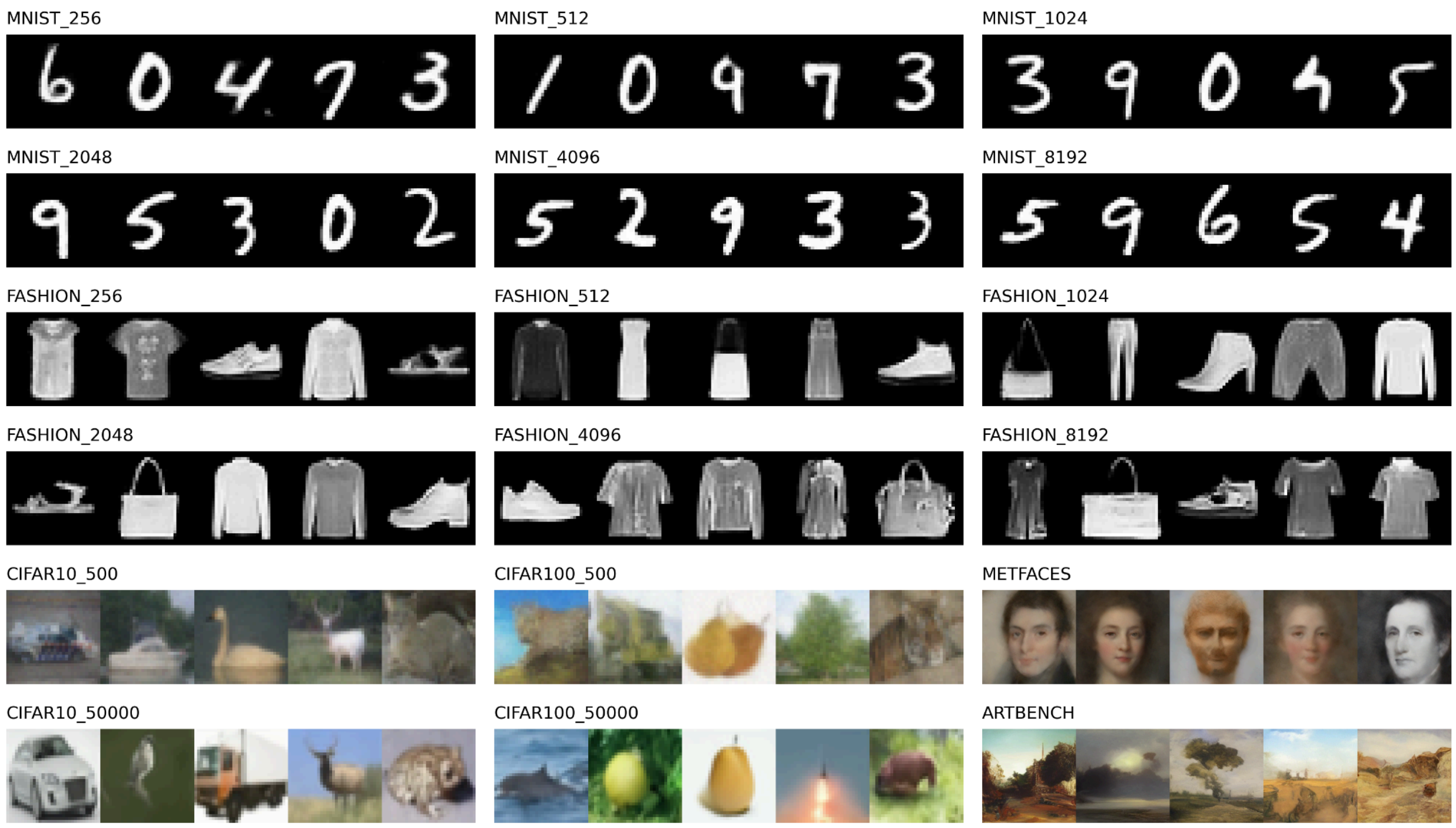

Supplementary Fig. 7. Additional samples from diffusion ensembles

We provide a selection of samples generated from some of the diffusion ensembles documented in Supplementary Table 2.

Supplementary Tables

Image Distribution	Color	Resolution	Autoencoder	Split Trained On	Data Augmentation	μ	σ
**MNIST**	Grayscale	32x32	No Autoencoder	Test	None	0.50	0.50
**MNIST (Binarized)**	Binary	32x32	No Autoencoder	Train	None	0.50	0.50
**Fashion-MNIST**	Grayscale	32x32	No Autoencoder	Test	Horizontal mirroring	0.50	0.50
**CIFAR-10**	Color	32x32	No Autoencoder	Train	Horizontal mirroring	0.50	0.25
**CIFAR-100**	Color	32x32	No Autoencoder	Train	Horizontal mirroring	0.50	0.25
**CelebA (BA)**	Color	128x128	Basic Autoencoder	Train	None	0.07, 0.17, -0.02, -0.02	0.28, 0.76, 0.17, 0.13
**CelebA (SD)**	Color	256x256	Stable-Diffusion-v1-5	Train	None	1.96	5.43
**MetFaces**	Color	256x256	Stable-Diffusion-v1-5	N/A	Horizontal mirroring	1.47	4.44
**ArtBench**	Color	256x256	Stable-Diffusion-v1-5	Train	Horizontal mirroring	1.35	4.56

Supplementary Table 1. Image distributions

MNIST (Binarized) is constructed by binarizing the pixel values using a threshold of 0.5. Duplicates are removed, resulting in an image distribution of 59984 images from an original image distribution of 60000. CelebA (BA) and CelebA (SD) differ in the autoencoder used to translate between latent and pixel spaces, and we also scale CelebA (SD) to 256x256. μ and σ are values used to uniformly shift and scale samples within an image distribution to help training. Some are hand picked while others are derived from the image distributions. For CelebA (BA), the shifting and scaling is per-channel as opposed to across the entire image tensor, hence four values are provided for both μ and σ.

Ensemble	Image Distribution	Training Set Size	Min Split Size	Max Split Size	Number of Units	Ensemble Size	Epochs Trained	Hidden Units	Dropout	Conditioning
**MNIST_256**	MNIST	256	112	147	256	22	85932	128x256x512x512	0	None
**MNIST_512**	MNIST	512	233	281	512	22	42966	128x256x512x512	0	None
**MNIST_1024**	MNIST	1024	485	540	1024	22	21472	128x256x512x512	0	None
**MNIST_2048**	MNIST	2048	963	1071	2048	22	10736	128x256x512x512	0	None
**MNIST_4096**	MNIST	4096	2004	2098	4096	22	5368	128x256x512x512	0	None
**MNIST_8192**	MNIST	8192	3980	4211	8192	22	2684	128x256x512x512	0	None
**FASHION_256**	Fashion-MNIST	256	112	147	256	22	85932	128x256x512x512	0	None
**FASHION_512**	Fashion-MNIST	512	233	281	512	22	42966	128x256x512x512	0	None
**FASHION_1024**	Fashion-MNIST	1024	485	540	1024	22	21472	128x256x512x512	0	None
**FASHION_2048**	Fashion-MNIST	2048	963	1071	2048	22	10736	128x256x512x512	0	None
**FASHION_4096**	Fashion-MNIST	4096	2004	2098	4096	22	5368	128x256x512x512	0	None
**FASHION_8192**	Fashion-MNIST	8192	3980	4211	8192	22	2684	128x256x512x512	0	None
**CIFAR10_500**	CIFAR-10	500	231	267	500	22	2162400	192x384x768x768	0.1	None
**CIFAR10_50000**	CIFAR-10	50000	24801	25170	50000	22	13200	192x384x768x768	0.1	None
**CIFAR100_500**	CIFAR-100	500	231	267	500	22	2162400	192x384x768x768	0.1	None
**CIFAR100_50000**	CIFAR-100	50000	24801	25170	50000	22	13200	192x384x768x768	0.1	None
**CELEBA_BA_32**	CelebA (BA)	712	326	379	32	22	1126400	128x256x512x512	0	None
**CELEBA_BA_512**	CelebA (BA)	10627	5274	5351	512	22	70400	128x256x512x512	0	None
**CELEBA_BA_8192**	CelebA (BA)	162770	78866	82296	8192	22	4400	128x256x512x512	0	None
**CELEBA_SD_32**	CelebA (SD)	712	326	379	32	22	1126400	128x256x512x512	0	None
**CELEBA_SD_512**	CelebA (SD)	10627	5274	5351	512	22	70400	128x256x512x512	0	None
**CELEBA_SD_8192**	CelebA (SD)	162770	78866	82296	8192	22	4400	128x256x512x512	0	None
**METFACES**	MetFaces	1336	667	669	744	24	144000	128x256x512x512	0	None
**ARTBENCH**	ArtBench	50000	24999	25001	2108	22	8800	192x384x768x768	0.1	None

Supplementary Table 2. Ensembles

Detailed information on the main 24 ensembles that we used to derive the power law relationships between CR and training set size. The hidden units column indicates the sizes of the channels on each layer of the U-Net. The number of units indicates how many units of training data are in the training set of the ensemble, or equivalently the number of counterfactual scenarios that can be constructed through ablation.

Ensemble	Image Distribution	Training Set Size	Min Split Size	Max Split Size	Number of Units	Ensemble Size	Epochs Trained	Hidden Units	Dropout	Conditioning
**MNIST_96_256**	MNIST	256	119	144	96	14	54684	128x256x512x512	0	None
**MNIST_96_512**	MNIST	512	247	280	96	14	39060	128x256x512x512	0	None
**MNIST_96_1024**	MNIST	1024	491	526	96	14	15620	128x256x512x512	0	None
**MNIST_96_2048**	MNIST	2048	991	1052	96	14	7182	128x256x512x512	0	None
**MNIST_96_4096**	MNIST	4096	1998	2082	96	14	3544	128x256x512x512	0	None
**MNIST_96_8192**	MNIST	8192	3998	4203	96	14	1740	128x256x512x512	0	None
**FASHION_96_256**	Fashion-MNIST	256	119	144	96	14	54684	128x256x512x512	0	None
**FASHION_96_512**	Fashion-MNIST	512	247	280	96	14	39060	128x256x512x512	0	None
**FASHION_96_1024**	Fashion-MNIST	1024	491	526	96	14	15620	128x256x512x512	0	None
**FASHION_96_2048**	Fashion-MNIST	2048	991	1052	96	14	7182	128x256x512x512	0	None
**FASHION_96_4096**	Fashion-MNIST	4096	1998	2082	96	14	3544	128x256x512x512	0	None
**FASHION_96_8192**	Fashion-MNIST	8192	3998	4203	96	14	1740	128x256x512x512	0	None
**MNIST_CLASS_256**	MNIST	256	110	140	256	22	85932	128x256x512x512	0	Class
**MNIST_CLASS_512**	MNIST	512	233	276	512	22	42966	128x256x512x512	0	Class
**MNIST_CLASS_1024**	MNIST	1024	473	536	1024	22	21472	128x256x512x512	0	Class
**MNIST_CLASS_2048**	MNIST	2048	979	1059	2048	22	10736	128x256x512x512	0	Class
**MNIST_CLASS_4096**	MNIST	4096	1983	2093	4096	22	5368	128x256x512x512	0	Class
**MNIST_CLASS_8192**	MNIST	8192	4010	4189	8192	22	2684	128x256x512x512	0	Class
**FASHION_CLASS_256**	Fashion-MNIST	256	110	140	256	22	85932	128x256x512x512	0	Class
**FASHION_CLASS_512**	Fashion-MNIST	512	233	276	512	22	42966	128x256x512x512	0	Class
**FASHION_CLASS_1024**	Fashion-MNIST	1024	473	536	1024	22	21472	128x256x512x512	0	Class
**FASHION_CLASS_2048**	Fashion-MNIST	2048	979	1059	2048	22	10736	128x256x512x512	0	Class
**FASHION_CLASS_4096**	Fashion-MNIST	4096	1983	2093	4096	22	5368	128x256x512x512	0	Class
**FASHION_CLASS_8192**	Fashion-MNIST	8192	4010	4189	8192	22	2684	128x256x512x512	0	Class
**CELEBA_TEXT_32**	CelebA (SD)	712	326	379	32	22	1126400	128x256x512x512	0	Text
**CELEBA_TEXT_512**	CelebA (SD)	10627	5274	5351	512	22	70400	128x256x512x512	0	Text
**CELEBA_TEXT_8192**	CelebA (SD)	162770	78866	82296	8192	22	4400	128x256x512x512	0	Text
**CELEBA_EPOCH_512**	CelebA (SD)	10627	5274	5351	512	22	16896	128x256x512x512	0	None
**CELEBA_EPOCH_8192**	CelebA (SD)	162770	78866	82296	8192	22	16896	128x256x512x512	0	None
**MNIST_BINARIZED**	MNIST (Binarized)	59984	5995	6002	190	20	2000	128x256x512x512	0	None
**MNIST_TRJ_324**	MNIST	324	143	172	324	16	64000 minibatches	128x256x512x512	0	None
**MNIST_TRJ_6241**	MNIST	6241	3053	3169	6241	16	64000 minibatches	128x256x512x512	0	None
**FASHION_TRJ_324**	Fashion-MNIST	324	143	172	324	16	64000 minibatches	128x256x512x512	0	None
**FASHION_TRJ_6241**	Fashion-MNIST	6241	3053	3169	6241	16	64000 minibatches	128x256x512x512	0	None

Supplementary Table 3. Ensembles for additional experimental conditions

Detailed information on the ensembles we trained to assess additional experimental conditions. MNIST_TRJ_324 and MNIST_TRJ_6241 are the ensembles from the trajectory plotted in Figures 6 and Supplementary Fig. 3 that were trained on the least and most amount of data respectively. 60 additional models in between were left out of this summary table. Similarly, there are 60 additional models between FASHION_TRJ_324 and FASHION_TRJ_6241 that are not documented in this table. Note that for these TRJ models we sampled minibatches with replacement, thus 64000 indicates the number of minibatches and not the number of epochs.

Ensemble	Ensemble FID (Disjoint split)	Ensemble FID (Same split)	Single Model FID (Disjoint split)	Single Model FID (same split)
**MNIST_256**	38.31	39.16	19.91	19.49
**MNIST_512**	34.75	35.18	14.26	13.25
**MNIST_1024**	32.13	32.79	14.91	15.78
**MNIST_2048**	20.16	20.60	10.93	11.40
**MNIST_4096**	4.39	4.53	9.76	10.07
**MNIST_8192**	3.43	3.62	13.26	13.04
**FASHION_256**	51.93	52.19	45.00	44.80
**FASHION_512**	45.27	45.83	35.51	35.97
**FASHION_1024**	37.11	37.87	18.76	19.51
**FASHION_2048**	21.86	22.63	19.51	20.64
**FASHION_4096**	12.96	13.82	23.88	24.77
**FASHION_8192**	16.46	17.28	40.71	41.62
**CIFAR10_500**	76.18	75.10	51.46	49.78
**CIFAR10_50000**	23.52	21.67	15.38	13.31
**CIFAR100_500**	86.11	83.19	57.54	55.75
**CIFAR100_50000**	30.70	27.87	17.14	14.67
**CELEBA_BA_32**	39.03	39.44	25.49	25.05
**CELEBA_BA_512**	60.80	61.82	35.24	36.07
**CELEBA_BA_8192**	14.78	15.37	31.67	32.90
**CELEBA_SD_32**	54.41	55.33	20.46	20.17
**CELEBA_SD_512**	50.92	52.19	21.68	22.41
**CELEBA_SD_8192**	10.27	10.57	14.61	15.03
**METFACES**	N/A	98.67	N/A	53.56
**ARTBENCH**	43.02	40.30	38.84	36.08

Supplementary Table 4. FIDs of diffusion ensembles and diffusion models on matched training sets

FID values of the ensembles from Supplementary Table 2. FIDs are calculated both using a split that is disjoint from the set used to train the ensembles as reference (disjoint split) and using a split that intersects the set used to train the ensembles as reference (same split). For each ensemble the FID attained by a single model that is trained on a matching training dataset is also reported.

	P-Value				Details		
**Image Distribution/ Experimental Condition**	**Euclidean Distance**	**OpenCLIP**	**LPIPS**	**DINOv2**	**Small Training Set Size**	**Large Training Set Size**	**Sample Size (N)**
**MNIST**	2.34E-20	3.63E-09	2.95E-16	2.62E-13	256	8192	(100, 100)
**Fashion-MNIST**	3.83E-05	9.58E-05	3.40E-08	3.69E-03	256	8192	(100, 100)
**CIFAR-10**	6.19E-15	5.25E-14	6.19E-15	6.19E-15	500	50000	(100, 25)
**CIFAR-100**	2.48E-14	2.10E-13	6.82E-15	6.63E-14	500	50000	(100, 25)
**CelebA(BA)**	1.03E-33	3.77E-29	1.69E-31	7.05E-23	712	162770	(100, 100)
**CelebA(SD)**	7.92E-34	7.24E-28	3.34E-34	2.53E-25	712	162770	(100, 100)
**MetFaces/ ArtBench**	6.04E-22	1.60E-22	4.63E-32	1.14E-03	1336	50000	(100, 100)
**MNIST Class Conditioned**	1.21E-24	7.21E-14	1.95E-23	7.64E-21	256	8192	(100, 100)
**Fashion-MNIST Class Conditioned**	4.33E-19	4.80E-15	5.87E-18	8.19E-12	256	8192	(100, 100)
**CelebA Text Prompt Conditioned**	3.85E-23	2.13E-22	9.48E-27	5.89E-20	712	162770	(100, 100)
**CelebA Fixed Epoch**	5.07E-13	1.30E-3	1.76E-12	4.74E-5	10627	162770	(100, 100)
**MNIST Fixed Proportion**	4.23E-24	3.13E-17	1.15E-20	3.59E-20	256	8192	(100, 100)
**MNIST Retrain**	4.80E-04	1.16E-05	2.39E-07	5.55E-06	256	1024	(100, 100)
**Fashion-MNIST Fixed Proportion**	5.60E-04	2.25E-07	1.14E-06	5.35E-05	256	8192	(100, 100)
**Fashion-MNIST Retrain**	1.27E-07	2.14E-05	1.46E-09	4.32E-04	256	1024	(100, 100)

Supplementary Table 5. Statistical significance of attribution decay

P-Values calculated using the one-sided Mann-Whitney U test to assess whether the distribution of CRs from the model trained on the smallest training set is larger than the distribution of CRs from the model trained on the largest training set in specific groups. This table is companion to Supplementary Fig. 2 and 5, with Supplementary Fig. 2 corresponding to the first 7 rows (main 24 ensembles) and Supplementary Fig. 5 corresponding to the last 8 rows (additional experimental conditions). The Mann-Whitney U test takes two distributions as input. The sizes of those distributions are given in the “Sample Size” column, where the first entry is the number of samples sampled from the ensemble trained on the smaller training set and the second entry is the number of samples sampled from the ensemble trained on the larger training set.

		Counterfactual Attributability				Similarity Based Retrievability			
		Euclidean Distance	Open CLIP	LPIPS	DINO v2	Euclidean Distance	Open CLIP	LPIPS	DINO v2
Counter factual Attribut ability	Euclidean Distance	1	0.472	0.59	0.426	0.074	-0.013	0.093	-0.048
	OpenCLIP	0.472	1	0.551	0.552	0.125	-0.082	0.068	-0.101
	LPIPS	0.59	0.551	1	0.535	0.157	-0.049	0.032	-0.109
	DINOv2	0.426	0.552	0.535	1	0.119	-0.093	0.007	-0.179
Similarity Based Retriev ability	Euclidean Distance	0.074	0.125	0.157	0.119	1	0.092	0.354	-0.022
	OpenCLIP	-0.013	-0.082	-0.049	-0.093	0.092	1	0.215	0.363
	LPIPS	0.093	0.068	0.032	0.007	0.354	0.215	1	0.148
	DINOv2	-0.048	-0.101	-0.109	-0.179	-0.022	0.363	0.148	1

Supplementary Table 6. Comparison between counterfactual attributability and similarity based retrievability

We compute the average Kendall rank correlation coefficients between attributability as measured by CRs and similarity based retrievability. Coefficients are also computed between different attributability measures that use different image distance metrics and different retrievability measures that use different image distance metrics.
