# Supplementary material for: Outputs of generative diffusion models are often unattributable
Source: Nat Commun. 2026 Aug 18;17:6974. doi: 10.1038/s41467-026-75667-5 (PMC13487234; doi:10.1038/s41467-026-75667-5)
Supplement: Supplementary file 2 [file 41467_2026_75667_MOESM2_ESM.pdf]

nature portfolio

Peer Review File

Outputs of Generative Diffusion Models are Often Unattributable

Corresponding Author: Dr Zheng Dai

This file contains all reviewer reports in order by version, followed by all author rebuttals in order by version.

Version 0:

Reviewer comments:

Reviewer #1

(Remarks to the Author)
In the study, the authors show that the outputs of diffusion generative models can be unattributable, specifically in a high data regime. Attributability of a generated image is defined as the largest change that can be induced in the image by omitting a single unit from the training data. This is an interesting paper with thorough experimental evaluations and control experiments. I thank the authors for that.

However, there are several limitations in the presentation, and some aspects could be explained more clearly. My comments are listed below.

1 - While the title refers broadly to generative models, the experiments focus on diffusion models. It would be helpful to clarify why diffusion models were chosen for this study and why other state of the art approaches such as visual autoregressive transformers (https://arxiv.org/abs/2404.02905) were not included. Either otehr generative models should be included or the title of the paper should be modified accordingly.

2 - A discussion of how these findings might generalize to other classes of generative models would strengthen the paper. In particular, it would be useful to explain why similar or different behavior might be expected in other model families.

3 - At the end of the introduction, it would be helpful to clearly list the main contributions and indicate which section corresponds to each contribution. Numbering of sections would be helpful.

4 - For newly introduced concepts such as counterfactual universe and counterfactual radius, adding illustrative figures could improve clarity and make the ideas more accessible to the reader.

5 - Please consider clarifying how the prompts are selected, as mentioned around line 55. A few examples would be helpful.

6 - In Figure 3A it would be helpful to clarify whether PCA is computed directly in image space. Additional details on whether applying PCA at the pixel level is meaningful and on the image resolution used for this analysis would improve understanding.

7 - The discussion of the code C around lines 276 to 282 could be clarified further to improve readability.

8 - In Figure 5 the generated images appear somewhat blurry. It may be useful to include additional samples from the diffusion ensemble and individual diffusion models. It could be that diffusion ensemble models emphasize more global structure. Please discuss whether this could affect the final FID values.

9 - The notion of attributability seems closely related to memorization. Including a discussion that situates this work in the broader literature on memorization in generative models would strengthen the paper.

10 - Relatedly, it would be interesting to discuss whether factors known to reduce memorization also tend to reduce attributability.

11 - How long were the models trained? Were they trained by fixing the number of iterations or epochs? If it's by iterations, then it would mean that models trained on fewer samples would have seen the same training image more times, and this could affect the attributability. Please explain.

(Remarks on code availability)
The code is only shown for a very specific evaluation case. It would be helpful to actually demonstrate for a few ensemble trainings (perhaps just for MNIST) and then compare them.

Reviewer #2

(Remarks to the Author)
What are the noteworthy results?
This manuscript investigates the fundamental limits of attributing generative model outputs to individual units of training data. The authors introduce a causal, counterfactual framework for attribution based on leave-one-out counterfactual universes and propose a novel ablation-based methodology that enables efficient removal of training data influence without retraining. Using diffusion ensembles, they empirically demonstrate a robust phenomenon they term attribution decay: as training set size increases, generated outputs become increasingly insensitive to the removal of any single training unit (image, person, or artist).

Key noteworthy contributions include:
1. A principled causal definition of attribution using counterfactuals and the Counterfactual Radius metric.
2. A scalable ablation framework based on diffusion ensembles that allows exact causal removal of training data units.
3. Extensive empirical evidence across datasets, conditioning regimes, and similarity metrics that attribution decays following an inverse power law with dataset size.

Overall, the results are clearly presented and experimentally well-supported.

Will the work be of significance to the field and related fields? How does it compare to the established literature? If the work is not original, please provide relevant references.

While prior work has focused on influence functions, gradient tracing, or similarity-based attribution (e.g., Koh & Liang 2017; Pruthi et al. 2020; Park et al. 2023; Mlodozeniec et al. 2024), this paper differs fundamentally in that it studies whether attribution is possible at all, rather than proposing a better attribution method.

The framing of unattributability as an emergent property of large-scale training is novel and clarifies why many existing attribution approaches succeed only when large subsets of data are removed. The work also provides an important counterpoint to studies showing memorization or training data extraction (e.g., Carlini et al., 2023), by emphasizing that such events are rare and do not contradict aggregate unattributability.

This manuscript meaningfully advances conceptual understanding in the literature and will likely shape future research directions.

Does the work support the conclusions and claims, or is additional evidence needed?
Are there any flaws in the data analysis, interpretation and conclusions? Do these prohibit publication or require revision?

The conclusions are generally well supported by the experimental evidence:
1. Attribution decay is demonstrated consistently across datasets, unit definitions, conditioning methods, and similarity metrics.
2. The ablation methodology is validated via retraining experiments at smaller scales.
3. Statistical tests are appropriate and clearly reported.
The claim that attribution becomes “practically impossible” is justified in an average-case and causal sense, though it may benefit from additional clarification (see Suggestions below).

Is the methodology sound? Does the work meet the expected standards in your field?

The methodology is innovative and largely sound:
1. The causal framing of attribution via counterfactuals is rigorous and well motivated.
2. The ablation-based construction is elegant and avoids known pitfalls of approximate influence methods.
3. Diffusion ensembles are convincingly shown to be viable generative models with comparable performance to standard diffusion models.

Is there enough detail provided in the methods for the work to be reproduced?

The paper provides substantial methodological detail, and the public release of code and data is commendable. The reporting of datasets, metrics, and statistical tests is clear. Reproducibility appears feasible for researchers with adequate computational resources.

Weakness

1. Interpretation of “Unattributability”
The manuscript occasionally risks being interpreted as claiming that no meaningful attribution is possible under any circumstances. It would be helpful to more explicitly distinguish causal leave-one-out attribution at the level of individual units (the focus here), from aggregate, subset-level, or approximate attribution methods that may still be useful in practice. Clarifying this distinction in the Discussion would reduce the risk of overgeneralization.

2. Dependence on Model Architecture
All experiments are conducted using diffusion ensembles. While retraining experiments partially address this, the conclusions may still be perceived as architecture-dependent. A more explicit discussion of how and why attribution decay should generalize beyond diffusion ensembles (e.g., to standard diffusion models or other generative architectures) would strengthen the claims.

3. Choice and Interpretation of Counterfactual Radius
CR is a worst-case measure over the counterfactual universe. While well motivated, some discussion of alternative summaries (e.g., quantiles or expected distances) would help readers understand whether attribution decay is driven by extreme or typical counterfactuals.

4. Lack of comparisons with other baseline methods
Can the author manage to compare their method with other baseline methods? And report some widely used metrics like Linear datamodeling score.

Overall
I believe the paper is suitable for publication in Nature Communications after minor to moderate revisions addressing the clarification points above.

(Remarks on code availability)
The public release of code and data is commendable.

However, it does not provide a README file with enough instructions for installing and running the application.

I did not run the code.

Version 1:

Reviewer comments:

Reviewer #1

(Remarks to the Author)
Thank you for addressing my concerns. I do not have any other major comments. Just a couple of minor comments:
On lines 381 and 388, there are empty blocks.
On line 295, 'data deduplication, which is generally thought to mitigate memorization', isn't it the opposite?

(Remarks on code availability)

Open Access This Peer Review File is licensed under a Creative Commons Attribution 4.0 International License, which permits use, sharing, adaptation, distribution and reproduction in any medium or format, as long as you give appropriate credit to the original author(s) and the source, provide a link to the Creative Commons license, and indicate if changes were made.
In cases where reviewers are anonymous, credit should be given to 'Anonymous Referee' and the source.
The images or other third party material in this Peer Review File are included in the article's Creative Commons license, unless indicated otherwise in a credit line to the material. If material is not included in the article's Creative Commons license and your intended use is not permitted by statutory regulation or exceeds the permitted use, you will need to obtain permission directly from the copyright holder.
To view a copy of this license, visit https://creativecommons.org/licenses/by/4.0/

We thank the reviewer for their thoughtful comments and for their suggestions. We have included our responses inline in red under each point. Line number references to where revisions have been made to address are also included. We believe our paper has been significantly strengthened as a result and we hope that our responses adequately address the concerns raised.

Reviewer #1 (Remarks to the Author):

In the study, the authors show that the outputs of diffusion generative models can be unattributable, specifically in a high data regime. Attributability of a generated image is defined as the largest change that can be induced in the image by omitting a single unit from the training data. This is an interesting paper with thorough experimental evaluations and control experiments. I thank the authors for that.

However, there are several limitations in the presentation, and some aspects could be explained more clearly. My comments are listed below.

1 - While the title refers broadly to generative models, the experiments focus on diffusion models. It would be helpful to clarify why diffusion models were chosen for this study and why other state of the art approaches such as visual autoregressive transformers (https://arxiv.org/abs/2404.02905) were not included. Either otehr generative models should be included or the title of the paper should be modified accordingly.

Diffusion models are highly capable generative models with widespread adoption, which makes them an important object of study. We also note that the effectiveness of the diffusion ensemble, which allows us to study attributability via ablation, also makes diffusion a particularly suitable class of models for our study. We have modified the text (lines 80-82) to include the latter statement.

An extension of this study to autoregressive models would require the invention of a similarly ablatable architecture in the autoregressive setting, which we view as a significant research challenge outside of the scope of the current work. Therefore to better reflect the experimental scope of our paper we have amended our title to “Outputs of Generative Diffusion Models are Often Unattributable” per the reviewer’s suggestion.

2 - A discussion of how these findings might generalize to other classes of generative models would strengthen the paper. In particular, it would be useful to explain why similar or different behavior might be expected in other model families.

We believe that attribution decay is primarily caused by redundancies in the training data, which we discuss in the discussion section. If this is correct, then the primary driver of attribution decay is a feature of the dataset and not of the model. Therefore we expect these redundancies in the training data would cause other model families to similarly exhibit attribution decay. We

have added this observation to the discussion section (lines 303-305) to strengthen the discussion.

3 - At the end of the introduction, it would be helpful to clearly list the main contributions and indicate which section corresponds to each contribution. Numbering of sections would be helpful.

We have added a list of our main contributions at the end of the introduction section (lines 42-54). While the editorial guidance is to not number the sections, we have listed the names of the relevant subsections for navigability.

4 - For newly introduced concepts such as counterfactual universe and counterfactual radius, adding illustrative figures could improve clarity and make the ideas more accessible to the reader.

We have added a new figure as Figure 2a to better illustrate the idea of the Counterfactual Universe and Counterfactual Radius for improved clarity.

5 - Please consider clarifying how the prompts are selected, as mentioned around line 55. A few examples would be helpful.

We have amended the text (line 142) to clarify that the details prompt selection can be found in the Supplementary Information. We have also amended the Supplementary Notes (lines 220-225) to provide example prompts for further clarity.

6 - In Figure 3A it would be helpful to clarify whether PCA is computed directly in image space. Additional details on whether applying PCA at the pixel level is meaningful and on the image resolution used for this analysis would improve understanding.

We have amended the Figure 3 legend to clarify that the PCA is indeed computed directly in pixel level image space, and that images are all scaled to 256x256x3 before applying PCA. The PCAs are therefore all presented at the same scale. The PCA provides insight into the geometric properties of the counterfactual universe, which we believe is meaningful, but has limitations as discussed in the text (lines 102-104).

7 - The discussion of the code C around lines 276 to 282 could be clarified further to improve readability.

We have amended the paragraph to clarify the exact specifications required for our code C, specifically that it can be any collection of binary strings of a given length as long as the number of 1s in each string is held constant throughout the code (lines 376-378).

8 - In Figure 5 the generated images appear somewhat blurry. It may be useful to include additional samples from the diffusion ensemble and individual diffusion models. It could be that

diffusion ensemble models emphasize more global structure. Please discuss whether this could affect the final FID values.

We have amended Figure 5 to include samples from individual diffusion models trained on the same datasets as the ensembles, and we have included additional samples generated by the diffusion ensembles in Supplementary Figure 7. We do not observe any significant difference in sample quality and global structure between the ensembles and individual models, with the exception of the ensemble and model trained on MetFaces, where the samples generated by the ensemble are significantly blurrier.

We note that MetFaces has the smallest image distribution out of those presented in Figure 5. In general, we observe that images generated by diffusion ensembles tend to be blurrier than those generated by individual models when the training dataset is small. This is reflected in the FID scores, where ensembles trained on very small datasets all have higher FIDs than those of individual diffusion models. This blurriness in small training data regimes can also be observed in Figures 3a and 4a. This behavior disappears in the large training data regime. We have amended the text to include this observation (lines 410-433).

9 - The notion of attributability seems closely related to memorization. Including a discussion that situates this work in the broader literature on memorization in generative models would strengthen the paper.

We have edited our discussion to note that the result we reference about training data copying comes from the memorization literature (line 275), and have added a discussion on how our results of attribution decay are congruent with the observations in the memorization literature that training dataset size has an influence on training data copying (lines 283-289). Memorization is a broad and heterogenous subject, so we have focused our discussion on the memorization literature relating to generative diffusion models. We note that the discussed results focus largely on copying, which is a concept based on image similarity, and therefore does not preclude the existence of other signals within generated samples that can be used to perform attribution, which our counterfactual analysis does.

10 - Relatedly, it would be interesting to discuss whether factors known to reduce memorization also tend to reduce attributability.

Given the analogous role of training data in both memorization and attributability, we would not be surprised if other factors known to reduce memorization would also reduce attributability. An interesting case is data deduplication, which would likely improve attributability since the counterfactual omission of a deduplicated sample is likely to induce a stronger effect on the generated sample. This ties into the existing discussion of what we conjecture to be the underlying cause of attribution decay. We expanded the discussion to include this observation (lines 289-305).

11 - How long were the models trained? Were they trained by fixing the number of iterations or epochs? If it's by iterations, then it would mean that models trained on fewer samples would have seen the same training image more times, and this could affect the attributability. Please explain.

The models were trained by varying the number of epochs to fix the number of iterations. Models begin producing visually coherent samples after a certain number of iterations rather than epochs, therefore iterations are the natural and less compute intensive choice. We have augmented Supplementary Tables 2 and 3 to report the number of epochs these models were trained on.

We thank the reviewer for highlighting this important factor to consider. We have now expanded our results to include an “Epoch Fixed” experimental condition where we train two ensembles on CelebA for an equal number of epochs. We found that attribution decay still persists under this new control condition ($p < 10^{-12}$ for geometric CRs and $p < 0.002$ for semantic CRs via the one-sided Mann-Whitney U test). We have added this result to the paper in Figure 3 along with a corresponding subsection (lines 174-188), and we have amended the Supplementary Tables to account for these new experiments.

Reviewer #1 (Remarks on code availability):

The code is only shown for a very specific evaluation case. It would be helpful to actually demonstrate for a few ensemble trainings (perhaps just for MNIST) and then compare them.

We have added a set of demo scripts that run through the entire training to CR measurement pipeline in our most recent code release, which we have also included as part of our submission.

We thank the reviewer for their thoughtful comments and for their suggestions. We have included our responses inline in red under each point under “Weaknesses” and under “Remarks on code availability”. Line number references to where revisions have been made to address the feedback are also included. We believe our paper has been significantly strengthened as a result and we hope that our responses adequately address the concerns raised.

Reviewer #2 (Remarks to the Author):

What are the noteworthy results?

This manuscript investigates the fundamental limits of attributing generative model outputs to individual units of training data. The authors introduce a causal, counterfactual framework for attribution based on leave-one-out counterfactual universes and propose a novel ablation-based methodology that enables efficient removal of training data influence without retraining. Using diffusion ensembles, they empirically demonstrate a robust phenomenon they term attribution decay: as training set size increases, generated outputs become increasingly insensitive to the removal of any single training unit (image, person, or artist).

Key noteworthy contributions include:

1. A principled causal definition of attribution using counterfactuals and the Counterfactual Radius metric.
2. A scalable ablation framework based on diffusion ensembles that allows exact causal removal of training data units.
3. Extensive empirical evidence across datasets, conditioning regimes, and similarity metrics that attribution decays following an inverse power law with dataset size.

Overall, the results are clearly presented and experimentally well-supported.

Will the work be of significance to the field and related fields? How does it compare to the established literature? If the work is not original, please provide relevant references.

While prior work has focused on influence functions, gradient tracing, or similarity-based attribution (e.g., Koh & Liang 2017; Pruthi et al. 2020; Park et al. 2023; Mlodozeniec et al. 2024), this paper differs fundamentally in that it studies whether attribution is possible at all, rather than proposing a better attribution method.

The framing of unattributability as an emergent property of large-scale training is novel and clarifies why many existing attribution approaches succeed only when large subsets of data are removed. The work also provides an important counterpoint to studies showing memorization or training data extraction (e.g., Carlini et al., 2023), by emphasizing that such events are rare and do not contradict aggregate unattributability.

This manuscript meaningfully advances conceptual understanding in the literature and will likely shape future research directions.

Does the work support the conclusions and claims, or is additional evidence needed?
Are there any flaws in the data analysis, interpretation and conclusions? Do these prohibit publication or require revision?

The conclusions are generally well supported by the experimental evidence:
1. Attribution decay is demonstrated consistently across datasets, unit definitions, conditioning methods, and similarity metrics.
2. The ablation methodology is validated via retraining experiments at smaller scales.
3. Statistical tests are appropriate and clearly reported.
The claim that attribution becomes “practically impossible” is justified in an average-case and causal sense, though it may benefit from additional clarification (see Suggestions below).

Is the methodology sound? Does the work meet the expected standards in your field?

The methodology is innovative and largely sound:
1. The causal framing of attribution via counterfactuals is rigorous and well motivated.
2. The ablation-based construction is elegant and avoids known pitfalls of approximate influence methods.
3. Diffusion ensembles are convincingly shown to be viable generative models with comparable performance to standard diffusion models.

Is there enough detail provided in the methods for the work to be reproduced?

The paper provides substantial methodological detail, and the public release of code and data is commendable. The reporting of datasets, metrics, and statistical tests is clear. Reproducibility appears feasible for researchers with adequate computational resources.

Weakness

1. Interpretation of “Unattributability”
The manuscript occasionally risks being interpreted as claiming that no meaningful attribution is possible under any circumstances. It would be helpful to more explicitly distinguish causal leave-one-out attribution at the level of individual units (the focus here), from aggregate, subset-level, or approximate attribution methods that may still be useful in practice. Clarifying this distinction in the Discussion would reduce the risk of overgeneralization.

We have expanded the discussion (lines 254-260) to more explicitly state that our findings apply specifically to unit level leave-one-out attribution, and that this differs from aggregate, subset-level, or approximate attribution methods. We also note that although our findings do not preclude the ability to perform these other kinds of practically useful attributions, further refinement along these lines will likely not yield methods that can perform unit level leave-one-out attributions, the implications of which we discuss throughout the remainder of the section.

2. Dependence on Model Architecture
All experiments are conducted using diffusion ensembles. While retraining experiments partially address this, the conclusions may still be perceived as architecture-dependent. A more explicit discussion of how and why attribution decay should generalize beyond diffusion ensembles (e.g., to standard diffusion models or other generative architectures) would strengthen the claims.

We believe that the primary cause of attribution decay is the redundancy of learnable features in large training datasets. This would imply that the main driver of attribution decay arises from the dataset rather than the model. Therefore, we would expect that these training data redundancies would cause other generative architectures to exhibit attribution decay as well. We have added this observation to the discussion section (lines 295-305) to strengthen the discussion.

3. Choice and Interpretation of Counterfactual Radius
CR is a worst-case measure over the counterfactual universe. While well motivated, some discussion of alternative summaries (e.g., quantiles or expected distances) would help readers understand whether attribution decay is driven by extreme or typical counterfactuals.

We have performed and included additional analysis where we measured the correlation between training set size and alternative summaries of the counterfactual universe. Specifically we considered the mean distance between a factual sample and the members of its counterfactual universe, the median distance (50th percentile), the 25th percentile, and the 75th percentile. We find that these alternative summaries also exhibit negative correlation to the training set size, although this relation diminishes at lower percentiles like the 25th. This analysis is presented in Supplementary Figure 6.

4. Lack of comparisons with other baseline methods
Can the author manage to compare their method with other baseline methods? And report some widely used metrics like Linear datamodeling score.

To demonstrate the viability of diffusion ensembles, we report the widely used Frechet Inception Distance and compare it against a selection of contemporary image generation methods.

The other methodological innovations like the Counterfactual Radius are ways to measure attributability and are not directly usable as methods for performing attribution. Importantly, Counterfactual Radiuses cannot be interpreted as perturbations to some univariate model output, which is what the Linear Datamodeling Score (LDS) was designed to and excels at evaluating.

However, we understand the need to contextualize the Counterfactual Radius in the landscape of related measures. To that end, we have performed and included additional analysis where we analyze the concordance between attributability as measured by our Counterfactual Radius metric and baseline retrievability measures that have been used in the literature. Here we define

retrievability as the image similarity between a generated sample and the training data, which is a notion that is similar to attributability and is used in existing literature, for example in the data extraction work of Carlini et al. that we've referenced. We find that there is some concordance between attributability measures and retrievability measures, which we report in Supplementary Table 6. Our approach is documented in the Supplementary Notes (lines 300-332). We emphasize that the Counterfactual Radius is a measure of attributability rather than a method for performing attribution, so these measures of concordance are not measures of performance, but rather a way to contextualize the Counterfactual Radius in the landscape of related measures.

Overall
I believe the paper is suitable for publication in Nature Communications after minor to moderate revisions addressing the clarification points above.

Reviewer #2 (Remarks on code availability):

The public release of code and data is commendable.

However, it does not provide a README file with enough instructions for installing and running the application.

I did not run the code.

We have added a detailed README in our most recent code release, which is included in our submission and adheres to the checklist requirements set forth by the journal.

REVIEWERS' COMMENTS

We appreciate the further remarks and comments, and have included our responses inline in red.

Reviewer #1 (Remarks to the Author):

Thank you for addressing my concerns. I do not have any other major comments. Just a couple of minor comments:
On lines 381 and 388, there are empty blocks.

These are formatting errors which we have now fixed. Thank you for flagging.

On line 295, 'data deduplication, which is generally thought to mitigate memorization', isn't it the opposite?

We have amended the line to the less ambiguous "data deduplication, which is generally thought to reduce memorization". The consensus in the literature is that the removal of duplicated features in datasets decreases the propensity of models to display memorization behavior.
